# Circulating MicroRNA Expression Levels Associated With Internet Gaming Disorder

**DOI:** 10.3389/fpsyt.2018.00081

**Published:** 2018-03-12

**Authors:** Minho Lee, Hyeyoung Cho, Seung Hyun Jung, Seon-Hee Yim, Sung-Min Cho, Ji-Won Chun, Soo-Hyun Paik, Yae Eun Park, Dong Huey Cheon, Ji Eun Lee, Jung-Seok Choi, Dai-Jin Kim, Yeun-Jun Chung

**Affiliations:** ^1^Catholic Precision Medicine Research Center, College of Medicine, The Catholic University of Korea, Seoul, South Korea; ^2^Integrated Research Center for Genome Polymorphism, College of Medicine, The Catholic University of Korea, Seoul, South Korea; ^3^Department of Microbiology, College of Medicine, The Catholic University of Korea, Seoul, South Korea; ^4^Cancer Evolution Research Center, College of Medicine, The Catholic University of Korea, Seoul, South Korea; ^5^Department of Psychiatry, Seoul St. Mary’s Hospital, College of Medicine, The Catholic University of Korea, Seoul, South Korea; ^6^Department of Biochemistry, College of Life Science and Biotechnology, Yonsei University, Seoul, South Korea; ^7^Center for Theragnosis, Biomedical Research Institute, Korea Institute of Science and Technology, Seoul, South Korea; ^8^Department of Biomedical Engineering, Sogang University, Seoul, South Korea; ^9^Department of Psychiatry, SMG-SNU Boramae Medical Center, Seoul, South Korea

**Keywords:** Internet gaming disorder, microRNA, biomarker, addiction, western blot

## Abstract

**Background:**

Addictive use of the Internet and online games is a potential psychiatric disorder termed Internet gaming disorder (IGD). Altered microRNA (miRNA) expression profiles have been reported in blood and brain tissue of patients with certain psychiatric disorders and suggested as biomarkers. However, there have been no reports on blood miRNA profiles in IGD.

**Methods:**

To discover IGD-associated miRNAs, we analyzed the miRNA expression profiles of 51 samples (25 IGD and 26 controls) using the TaqMan Low Density miRNA Array. For validation, we performed quantitative reverse transcription PCR with 36 independent samples (20 IGD and 16 controls).

**Results:**

Through discovery and independent validation, we identified three miRNAs (hsa-miR-200c-3p, hsa-miR-26b-5p, hsa-miR-652-3p) that were significantly downregulated in the IGD group. Individuals with all three miRNA alterations had a much higher risk of IGD than those with no alteration [odds ratio (OR) 22, 95% CI 2.29–211.11], and the ORs increased dose dependently with number of altered miRNAs. The predicted target genes of the three miRNAs were associated with neural pathways. We explored the protein expression of the three downstream target genes by western blot and confirmed that expression of GABRB2 and DPYSL2 was significantly higher in the IGD group.

**Conclusion:**

We observed that expressions of hsa-miR-200c-3p, hsa-miR-26b-5p, and hsa-miR-652-3p were downregulated in the IGD patients. Our results will be helpful to understand the pathophysiology of IGD.

## Introduction

Addictive use of the Internet and Internet-based games is not just a social phenomenon in countries with extensive Internet access infrastructure, but a potential psychiatric disorder termed Internet gaming disorder (IGD) (1–3). According to epidemiological reports, prevalence rates of IGD in adolescents vary across countries, ranging from 0.8 to 26.7% (4). Particularly, studies show prevalence rates above 10% in adolescents in many Asian countries such as South Korea, China, Taiwan, Hong Kong, and Singapore (4). IGD is associated with impairment in cognition, psycho-social relationships, and daily life; for example, declining academic or occupational performance (4–7). IGD is now included in Section III (Conditions for Further Study) of the fifth revision of the Diagnostic and Statistical Manual of Mental Disorders (DSM-V) (8). However, in spite of its clinico-social importance, little is known about the molecular genetic mechanism behind IGD.

Recent large-scale twin studies have suggested a genetic background to IGD (9, 10). Vink et al. investigated individual differences in compulsive Internet use with 5,247 monozygotic and dizygotic adolescent twins in the Netherlands Twin Register and reported that 48% of the differences were explained by genetic factors (9). Li et al. observed 825 pairs of Chinese adolescent twins and reported that genetic factors explained 58–66% of the differences (10). Accordingly, polymorphisms of the genes involved in neurotransmission, cognition, and attention such as dopamine receptor D2 gene (*DRD2*), catecholamine-O-methyltransferase gene (*COMT*), serotonin transporter gene (*5HTTLPR*), and cholinergic receptor nicotinic alpha 4 gene (*CHRNA4*) have been reported to be significantly associated with Internet addiction (11–13). Recently, Kim et al. screened variants of more than 100 candidate genes related to production, action, and metabolism of neurotransmitters by next generation sequencing analysis and reported that rs2229910 of *NTRK3* gene is associated with IGD (14).

In addition to the genetic factors, it is also well known that neurobehavioral phenotypes are epigenetically controlled by non-coding RNAs including microRNAs (miRNAs) (15, 16). miRNAs are small non-coding single-stranded RNA molecules (approximately 20–23 nucleotides in length), that negatively regulate expression of protein-coding genes by degrading mRNAs and play a critical role in the pathophysiological process of diverse diseases (17). Lines of evidence have demonstrated that miRNAs are abundant in the human central nervous system (CNS) and act to fine tune the expression levels of their target genes, which are involved in the development and maturation of CNS system (15). Indeed, recent studies have revealed that miRNA expression profiles are altered in brain tissue of patients with psychiatric disorders, suggesting that their expression profiles could be biomarkers for psychiatric disorders (15, 16, 18). For example, through postmortem analysis, Lopez et al. reported that expression of miR-1202, which regulates the expression of metabotropic glutamate receptor-4 gene and predicts the response to antidepressant, was downregulated in prefrontal cortex tissues of major depression disorder patients (19). In terms of biomarker screening, this approach has a clear limitation because performing a biopsy of CNS tissue for screening is impossible. Since miRNAs can be detected in blood (plasma or serum), circulating miRNAs have a definite advantage as non-invasive biomarkers in neuropsychiatric disorders. However, to date, there have been no studies about circulating miRNA profiles in IGD. Better understanding of circulating miRNA expression profiles could help to clarify the mechanism of IGD development and facilitate clinical translation.

In this study, we aimed to identify IGD-associated miRNA markers by observing differentially expressed plasma miRNAs between the IGD and control groups and explored their biological implications.

## Materials and Methods

### Study Subjects

We surveyed 3,166 teenagers (aged 12–18 years) using DSM-V IGD scoring. Among them, 251 (168 males and 83 females) were diagnosed as IGD according to the DSM-V criteria (8). A total of 91 individuals (49 IGDs and 42 controls) provided the informed consent for this study. Among them, four individuals were excluded according to the exclusion criteria. Finally, 87 individuals (45 IGD subjects and 42 healthy control individuals) were enrolled for this study. Among them, 51 participants (25 IGD patients and 26 controls) were recruited as the discovery set from 2014 to 2016. The other 36 participants (20 IGD patients and 16 controls) were recruited as the independent validation set from 2016. All participants were Korean individuals, enrolled from Seoul St. Mary’s Hospital (Seoul, South Korea) and Seoul National University Boramae Hospital (Seoul, South Korea). All participants underwent a structured interview by a psychiatrist based on the Korean Kiddie Schedule for Affective Disorders and Schizophrenia (K-SADS-PL) (20). All participants completed the Block Design and Vocabulary subtests of the Korean-Wechsler Intelligence Scale for Children, 4th edition (K-WISC-IV) (21). Impulsiveness were assessed by Barratt Impulsiveness Scale (BIS) (22). Behavioral Inhibition System (BInS) and Behavioral Activation System (BAS) scales were measured to assess personality dimension (23). Exclusion criteria included past or current major medical disorders (e.g., diabetes mellitus), neurological disorder (e.g., seizure disorders, head injury), psychiatric disorders (e.g., major depressive disorder, anxiety disorders), mental retardation, or any substance abuse (e.g., tobacco, cannabis, alcohol). The general characteristics of the study subjects are summarized in Table 1. This study was approved by the Institutional Review Board of the Catholic University Medical College of Korea (MC16SISI0120). All participants and their parents gave written informed consent.

**Table 1:** General characteristics of the study subjects.

	Discovery			Validation			Combined		
			
	Control	IGD	*P*-value	Control	IGD	*P*-value	Control	IGD	*P*-value
*N*	26	25		16	20		42	45	
**Age (years)**									
Median (min–max)	13 (12–17)	13 (12–15)	0.759	15 (13–18)	14.5 (12–18)	0.628	14 (12–18)	14 (12–18)	0.509
**Weekly Internet gaming hours (h)**									
Median (min–max)	5.25 (2–17)	18 (6–46)	1.27E−6^a^	5.5 (2–23)	8 (1–112)	0.374	5.5 (2–23)	14 (1–112)	1.63E−5^a^
**Monthly household income (million KRW)**									
Median (min–max)	5 (1–9)	3 (1–9)	0.588	4 (4–4)	2 (2–2)	1.000	5 (1–9)	3 (1–9)	0.460
**Education (years)**									
Median (min–max)	8 (7–9)	8 (7–9)	0.584	12 (12–12)	6 (6–13)	0.305	8 (7–12)	8 (6–13)	0.269
**K-WISC: block design**									
Median (min–max)	10.5 (4–17)	10 (4–16)	0.544	10 (3–16)	12.5 (4–15)	0.125	10 (3–17)	11 (4–16)	0.598
**K-WISC: vocabulary**									
Median (min–max)	9 (5–17)	7 (5–13)	0.174	9.5 (8–15)	11.5 (5–15)	0.595	9 (5–17)	9 (5–15)	0.527
**KS**									
Median (min–max)	24 (17–36)	37 (22–51)	3.81E−6^a^	29 (17–34)	59 (22–108)	1.2E−5^a^	25 (17–36)	40 (22–108)	2.05E−10^a^
**BIS**									
Median (min–max)	63 (35–75)	67.5 (45–81)	0.080	61 (45–79)	63 (32–82)	0.835	62 (35–79)	65 (32–82)	0.240
**BAS**									
Median (min–max)	31 (15–40)	31 (13–51)	0.558	36.5 (22–48)	34 (27–52)	1.000	32 (15–48)	34 (13–52)	0.637
**BInS**									
Median (min–max)	18 (10–26)	17.5 (13–27)	0.642	18.5 (12–25)	20 (13–21)	0.138	18 (10–26)	19 (13–27)	0.302

*IGD, Internet gaming disorder patients; KS, Korean Internet Addiction Proneness Scale; BIS, Barratt Impulsivity Scale; BAS, Behavioral Activation System; BInS, Behavioral Inhibition System; KRW, Korean Won*.

*^a^*P* < 0.05 (Mann–Whitney–Wilcoxon test)*.

### TaqMan Low Density miRNA Array (TLDA) Experiments

Peripheral blood was collected from each participant and transferred to the laboratory within 4 h to minimize the blood cell lysis. The specimen was centrifuged at 3,000 rpm for 10 min at room temperature. Then, supernatant (plasma layer) was collected without contaminating the blood cells. Circulating miRNAs were extracted using TaqMan miRNA ABC Purification Kit (Human Panel A; Thermo Fisher Scientific, Waltham, MA, USA) according to the manufacturer’s instruction. In brief, 50 µL of plasma sample and 100 µL of ABC buffer were mixed. After hybridization with target-specific anti-miRNA magnetic beads, bounded circulating miRNAs were eluted from the beads with 100 µL of elution buffer. In the discovery phase, 381 miRNAs were examined from 51 plasma samples (25 IGDs and 26 controls) using the TaqMan miRNA ABC Purification Kit (Human Panel A; Thermo Fisher Scientific, Waltham, MA, USA) according to the manufacturer’s instructions. Megaplex reverse transcription and pre-amplification reactions were performed to increase the quantity of cDNA for miRNA expression analysis using MegaplexPreAmp Primers Human Pool A and TaqManPreAmp Master Mix (Thermo Fisher Scientific). The TLDA panel A v2.0 (Thermo Fisher Scientific) was run on the ViiA7 real-time PCR system (Thermo Fisher Scientific) to evaluate expression of the miRNAs. Raw data were processed using ExpressionSuite Software v1.0.4 (Thermo Fisher Scientific) to determine Ct values for each miRNA.

### Data Analysis for TLDA

We first measured threshold cycles (Ct value) of each miRNA. miRNAs with a Ct value >35 were considered as undetectable and excluded from subsequent analysis. All Ct values were normalized to the Ct value of miR-374b (ΔCt value), one of the most stably expressed miRNAs circulating in human plasma (24). A log2 fold-change ratio (ΔΔCt value) of expression was calculated using mean values of control samples as a calibrator in the HTqPCR package in Bioconductor (25). The relative quantification (RQ) of each miRNA target was defined as 2^−ΔΔCt^. For hypothetical testing of the difference in expression between two groups, we applied surrogate variable analysis (SVA) to capture heterogeneities such as batch effects in the experiments using the *sva* package in Bioconductor (26). miRNAs with a *P-*value <0.05 were considered to be significantly different between two groups.

### Gene Set Enrichment Analysis

For gene set enrichment analysis, we used ToppFun in ToppGene Suite (27) to infer significantly enriched Gene Ontology (GO) (28) terms, pathway, and disease terms. As the input for this approach, we used 1,230 predicted target genes of the candidate miRNAs. Pathway analysis was used to find significant pathways of the predicted target genes according to KEGG, BioCarta, Reactome, GeneMAPP, and MSigDBin the ToppGene pathways. The significance of functional enrichment terms was determined based on the Bonferroni-adjusted *P*-value.

### Quantitative Reverse Transcription PCR (qRT-PCR) Validation and Replication

To validate the 10 miRNAs that were differentially expressed in the discovery stage, qRT-PCR was performed using the TaqMan MicroRNA Assay (miR-15b-5p, #000390; miR-26b-5p, #000407; miR-29b-3p, #000413; miR-125b-5p, #000449; miR-200c-3p, #002300; miR-337-5p, #002156; miR-411-5p, #001610; miR-423-5p, #002340; miR-483-5p, #002338; and miR-652-3p, #002352) and the ViiA7 system (Life Technologies) according to the manufacturer’s protocol. Ten nanograms of total RNA was converted to first-strand cDNA with miRNA-specific primers using the TaqMan MicroRNA Reverse Transcription Kit (#4366596, Life Technologies), followed by real-time PCR with TaqMan Probes. The RQ of each miRNA was defined as 2^−ΔCt^, where ΔCt is the difference in threshold cycles for the sample in question, normalized against the endogenous miRNA (miR-374b-5p, #001319). All PCR reactions were carried out in triplicate, and their Ct values were averaged. We calculated a log2 fold-change ratio (ΔΔCt) of each miRNA in the same way as in the array-based analysis. A non-parametric Mann–Whitney–Wilcoxon test was performed to test the differences in expression levels of miRNAs in two groups with a threshold *P*-value of 0.05.

### Western Blot Analysis

Each serum sample was first depleted of the top 14 high-abundance proteins (albumin, immunoglobulin G, immunoglobulin A, serotransferrin, haptoglobin, alpha-1 antitrypsin, fibrinogen, alpha-2 macroglobulin, alpha-1 acid glycoprotein, immunoglobulin M, apolipoprotein A-I, apolipoprotein A-II, complement C3, and transthyretin) using the MARS-14 column (4.6 × 50 mm, Agilent Technology, Santa Clara, CA, USA) prior to western blot analysis. The unbound fraction obtained from the MARS-14 column was concentrated using an Amicon Ultracel-3 centrifugal filter (3 kDa cutoff), and then the protein concentration was determined using the bicinchoninic acid method. The same amounts (from 10 to 30 µg) of control and IGD serum samples were separated on a 4–20% Mini-PROTEAN TGX precast gel (Bio-Rad, CA, USA) and transferred to a polyvinylidene difluoride membrane. Next, the membrane was blocked in TBS-T (190 mM NaCl, 25 mM Tris–HCl, pH 7.5, and 0.05% Tween 20) with 5% non-fat dry milk at room temperature for 30 min. The membranes were then incubated with primary antibodies against DPYSL2 (1:500, Novus Biologicals, Littleton, CO, USA), GABRB2 (1:1000, Abcam, Cambridge, MA, USA), and CNR1 (1:100, Santa Cruz Biotechnology, Inc., Santa Cruz, CA, USA), DUSP4 (1:500, MybioSource, San Diego, CA, USA), and PI15 (1:500, MybioSource, San Diego, CA, USA) in TBS-T with 5% non-fat dry milk at 4°C overnight, and then with appropriate secondary antibodies either bovine anti-mouse (1:1,000, Santa Cruz Biotechnology) or goat anti-rabbit (1:1,000, Cell Signaling, Beverly, MA, USA) conjugated to horseradish peroxidase at room temperature for 1 h. Signal detection was performed using chemiluminescence with ECL reagent (GE healthcare, Piscataway, NJ, USA). We quantified the western blot results using the TotalLab 1D analysis software (Non-linear Dynamics, Newcastle upon Tyne, UK). Then, the densitometry ratio value was calculated by dividing the densitometry value of each sample as described elsewhere (29). As a control for normalization, a serum sample pooled from 46 IGD and control samples was used for every experiment. Statistical significance was determined using a non-parametric Mann–Whitney–Wilcoxon test with a threshold *P*-value of 0.05.

## Results

### Characteristics of the Study Subjects

The demographic and clinical features of the study subjects are shown in Table 1. When we compared the IGD and control groups according to the Korean Internet Addiction Proneness Scale (*K*-Scale) as described elsewhere (20, 30), the IGD group showed a significantly higher median K-Scale value than the control group (37 vs. 24, *P* = 3.81 × 10^−6^) (Table 1). Median weekly time spent on Internet gaming in the IGD group was significantly longer than that of controls (18 vs. 5.25 h, *P* = 1.27 × 10^−6^). Whereas there was no significant difference between two groups in age, monthly household income, duration of education, block design, and vocabulary subtest results of the K-WISC, BIS, BInS, and BAS.

### Differentially Expressed miRNAs Between IGD and Controls

**Figure 1:**
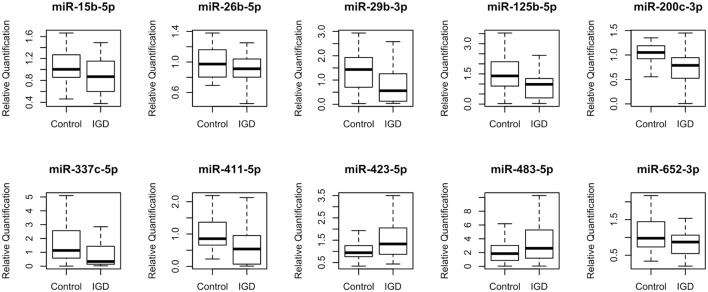
Relative expression levels of 10 differentially expressed miRNAs. Relative quantification (RQ) was normalized to miR-374b-5p.

To discover IGD-associated miRNAs, we adopted a two-step (discovery and independent validation) approach. The study design and overall strategy are illustrated in Figure S1 in Supplementary Material. In the discovery stage, we analyzed miRNA expression profiles of 51 samples (25 IGDs and 26 controls) using the miRNA array containing 384 miRNAs. Expression levels of 10 miRNAs were found to be significantly different between the IGD and control groups (Table 2). Relative expression levels of these 10 miRNAs are shown in Figure 1. Among them, two (hsa-miR-423-5p and hsa-miR-483-5p) were upregulated and eight (hsa-miR-15b-5p, hsa-miR-26b-5p, hsa-miR-29b-3p, hsa-miR-125b-5p, hsa-miR-200c-3p, hsa-miR-337c-5p, hsa-miR-411-5p, and hsa-miR-652-3p) were downregulated in the IGD group.

**Table 2:** Differentially expressed microRNAs (miRNAs) and fold changes.

miRNA	Discovery		Validation		Combined	
			
	*P*-value	Fold change	*P*-value	Fold change	*P*-value	Fold change
hsa-miR-15b-5p	0.033	0.829	0.694	1.119	0.381	0.947
hsa-miR-26b-5p^a^	0.008	0.871	0.049	0.841	0.013	0.857
hsa-miR-29b-3p	0.005	0.400	0.560	1.187	0.089	0.647
hsa-miR-125b-5p	0.021	0.582	0.290	0.950	0.069	0.723
hsa-miR-200c-3p^a^	0.011	0.336	0.003	0.542	2.93 × 10^−5^	0.415
hsa-miR-337c-5p	0.009	0.385	0.582	0.872	0.020	0.553
hsa-miR-411-5p	0.004	0.322	0.336	1.282	0.158	0.595
hsa-miR-423-5p	0.026	1.387	0.189	0.955	0.518	1.175
hsa-miR-483-5p	0.018	1.861	0.765	1.413	0.211	1.647
hsa-miR-652-3p^a^	0.019	0.715	0.049	0.877	0.011	0.782

*^a^miRNAs significantly altered in both discovery and validation sets in a consistent manner*.

### qRT-PCR Validation of the Candidate miRNAs

To validate the 10 candidate miRNAs, we performed qRT-PCR with an independent validation set (20 IGDs and 16 controls) (Table S1 in Supplementary Material). Three of these miRNAs (hsa-miR-200c-3p, hsa-miR-26b-5p, and hsa-miR-652-3p) were significantly downregulated in the IGD group of the validation set (Table 2). Three other miRNAs (hsa-miR-337c-5p, hsa-miR-125b, and hsa-miR-423-5p) were also downregulated in the IGD group but not significantly. Remaining four miRNAs (hsa-miR-15b-5p, hsa-miR-29b-3p, hsa-miR-411-5p, and hsa-miR-423-5p) were expressed oppositely in the validation set. When we combined the discovery and validation sets (a total of 45 IGD subjects and 42 controls), the three validated miRNAs were consistently significant (Table 2). Detailed information, chromosomal locations, mature sequences, and expression levels in the CNS of these three miRNAs are available in Table S2 in Supplementary Material.

### Synergistic Effect of Simultaneous Alteration of the Three miRNAs on IGD Risk

**Figure 2:**
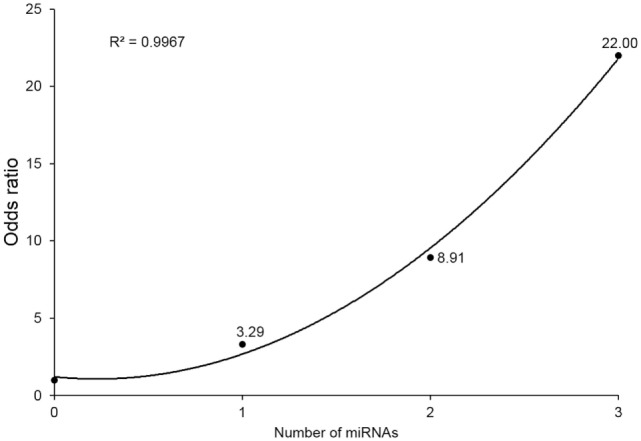
Odds ratios (ORs) by number of downregulated microRNA (miRNA) markers. Values above point estimates are the ORs (95% confidence interval).

To evaluate the combined effect of the three miRNAs, we observed the odds ratios (ORs) of the four subgroups (with 0, 1, 2, or 3 miRNA alterations). miRNA alteration was defined by the RQ value as described in Section “Materials and Methods.” Because all three miRNAs markers were downregulated in the IGD group, a miRNA whose RQ value was below one was defied as altered one. Detailed information of each study subject’s RQ value for the three miRNAs is available in Table S3 in Supplementary Material. For each subgroup, odds were calculated as the ratio of number of controls to that of IGDs, then each OR was calculated by dividing odds of each subgroup by odds of the subgroup without any miRNA alterations. Individuals with three miRNA alterations showed a risk 22 times higher than those without any miRNA alteration (OR 22, 95% CI 2.29–211.11). ORs showed an increasing trend with the number of altered miRNAs from 0 to 3 (*r*^2^ = 0.996) (Figure 2).

### GO and Pathway Analysis of Target Genes of the Candidate miRNAs

To gain insight into the functions of the three miRNA markers significantly downregulated in the IGD group, their target genes were predicted using the miRWalk 2.0 database (31). A total of 1,230 genes were consistently predicted as downstream targets by four algorithms (miRWalk, miRanda, RNA22, and Targetscan) using the miRWalk database (32–34) (Table S4 in Supplementary Material). Gene set enrichment analysis using ToppFun in ToppGene Suite showed that the target genes of those miRNAs were significantly associated with neural development pathways such as “Axon guidance” and GO terms such as “neurogenesis” (Table S5 in Supplementary Material).

### Expression of the Predicted Target Genes

**Figure 3:**
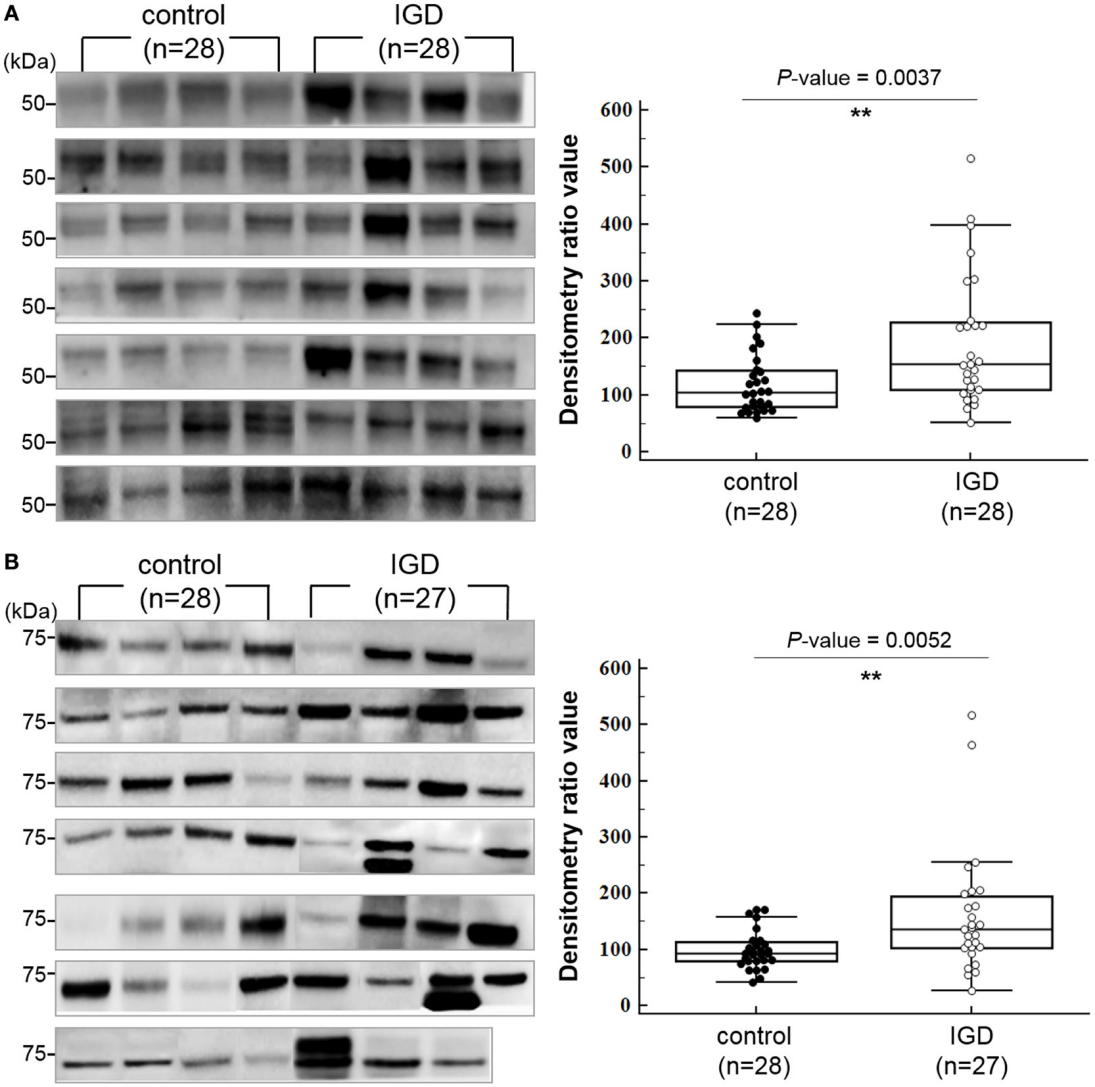
Western blot images and box-dot-plots showing expression of **(A)** DPYSL2 and **(B)** GABRB2. Both DPYSL2 and GABRB2 proteins exhibited significant differences in their expression levels between the Internet gaming disorder (IGD) and control samples (*P*-value <0.05). The two proteins were expressed at higher levels in the IGD samples.

Among the downstream target genes of the three miRNAs, 140 were predicted simultaneously for two or more miRNAs (Table S4 in Supplementary Material). To explore whether their protein expressions levels of the downstream target genes are different between the IGD and control groups, we selected 2 genes (*DUSP4* and *PI15*), which are predicted as downstream targets of all 3 miRNAs and additional 3 genes (*GABRB2, DPYSL2*, and *CNR1*) from those predicted for 2 miRNAs and performed western blot analysis with the plasma samples from 28 IGDs and 28 controls available for the experiment. We compared the expressions of the five targets between the IGD and control groups by measuring the band intensity and area as described elsewhere (29). Among them, the expression levels of DPYSL2 (28 IGDs and 28 controls, *P* = 0.0037) and GABBR2 (27 IGDs and 28 controls, *P* = 0.0052) were significantly higher in the IGD group (Figure 3). However, we could not observe differential expressions of CNR1 (*P* = 0.0853), DUSP4 (*P* = 0.5443), and PI15 (*P* = 0.6346).

## Discussion

It has been reported that miRNAs are involved in neuronal development (35, 36), and differential expression of brain miRNAs are observed in psychiatric diseases such as schizophrenia (37). Therefore, it is plausible that circulating miRNA profiles could be useful biomarkers for IGD. Circulating miRNAs have been suggested as biomarkers for diverse neuropsychiatric disorders (38–40); however, the molecular mechanisms behind IGD development are still largely unknown despite its clinical and social importance. Specifically, there have been no studies on IGD-associated miRNAs. The aim of this study was twofold. First, we attempted to discover plasma miRNAs associated with IGD. Second, we evaluated the biological implication of the miRNA candidates by exploring protein expression and GO of downstream target genes. Through genome-wide screening of miRNA expression profiles and downstream validation of the candidates, we discovered that expression of three miRNAs (hsa-miR-200c-3p, hsa-miR-26b-5p, and hsa-miR-652-3p) was significantly lower in IGD patients than controls. Although the expression patterns of other seven miRNA candidates were not replicated in the validation, it can be false negative due to small sample size in this study. To our knowledge, this is the first report on the possibility that blood miRNA expression profiles could be useful biomarkers for IGD. Combination of the three miRNA markers could serve as a minimally invasive tool for early identification of people at risk of IGD.

The miRNAs identified in this study have been reported to be involved in diverse neuropsychiatric disorders. Expression of hsa-miR-200c in blood has been reported to be downregulated in several psychiatric disorders such as schizophrenia (41) and major depressive episodes (42). miR-200c was reported to be more highly expressed in synaptic fractions than in total forebrain (43) and also to be associated with neuronal cell death (44). Based on these previous reports, miR-200c is involved in neurodevelopment and can be associated with neuropsychiatric disorders if its expression is perturbed. Several studies have suggested association between miR-652 and risk of neuropsychiatric disorders. Similar to our approach, to identify blood biomarkers for schizophrenia, Lai et al. carried out TLDA analysis with schizophrenia patients and normal controls, and found that seven miRNAs including hsa-miR-652 were differentially expressed in schizophrenia patients (45). In the subsequent study, they designed a prediction model using the miRNA expression data and successfully distinguished schizophrenia from normal control (46). Altered expression of hsa-miR-652 was also observed in alcoholics (47). Hsa-miR-26b was found to be activated during neuronal cell differentiation (48). Perkins et al. reported that hsa-miR-26b was downregulated in the prefrontal cortex of schizophrenia patients (49).

Although there is no direct evidence to support the relationship between the perturbed expression of these miRNAs and the pathophysiology of IGD, we can infer that dysregulation of these miRNAs may be associated with the pathophysiology of IGD based on various previous reports on the downstream genes we predicted. Some of the downstream genes of the three miRNAs such as *GABRB2, CNR1, NRXN1*, and *DPYSL2* are reported to be associated with neuropsychiatric disorders. Gamma-aminobutyric acid (GABA) is a major inhibitory neurotransmitter in the CNS. Dysregulation of the GABA receptor, is implicated in neuropsychiatric disorders including addiction, anxiety, and depression (50), which are also the main features of IGD (8). Genetic polymorphisms in GABA receptor genes are reported to be associated with alcohol addiction and schizophrenia (51, 52). Dihydropyrimidinase-like 2 (DPYSL2) is a member of the collapsin response mediator protein family, which plays a role in microtubule assembly, synaptic signaling, and regulation of axonal growth. Consequently, this molecule has been suggested as a biomarker for psychiatric disorders (53, 54). Polymorphism in the *DPYSL2* gene was also reported to be associated with alcohol use disorder (55). Previous reports and our data suggest that overexpression of GABRB2 and DPYSL2, downstream targets of the downregulated miRNAs, has implications for the pathogenesis of neuropsychiatric disorders including IGD. Cannabinoid receptor type 1 (CNR1) is a presynaptic heteroreceptor that modulates neurotransmitter release and disturbances in cannabinoid signaling are associated with various neuropsychiatric disorders (56). Genetic polymorphism of *CNR1* gene is known to be associated with substance dependence in Caucasians (57). In a rat model, activation of ventral hippocampus CNR1 disrupts normal social behavior and cognition (58). Genetic alteration in the NRXN family is known to be involved in diverse neuropsychiatric disorders including addiction (59).

To examine the biological implication of the three miRNA candidates in a more direct way, we explored protein expression of their downstream target genes. Due to the limited availability of plasma samples, of the 140 common candidates (predicted as downstream of 2 or more miRNAs), we examined 5 targets (GABRB2, DPYSL2, CNR1, DUSP4, and PI15) by western blot and confirmed that expression of GABRB2 and DPYSL2 was significantly higher in the IGD group. Previous reports and our data suggest that overexpression of GABRB2 and DPYSL2, downstream targets of the downregulated miRNAs, may have implications for the pathogenesis of neuropsychiatric disorders including IGD. The results of GO and pathway analysis of neural development pathways also support the neurobiological implication of the miRNA markers. Another interesting finding was the synergistic effect of simultaneous alteration of the miRNAs. Individuals with downregulation of all 3 miRNAs showed 22 times higher risk than those with no downregulation, and the ORs increased in a dose-dependent manner. Although CI for these three alterations were wide due to limited sample size, the clear positive correlation (*r*^2^ = 0.996) supports the synergistic effect of the three miRNAs.

Although we did discover the IGD-associated miRNA markers and individuals with all three miRNA alterations had a risk 22 times higher than those without any miRNA alterations, there are several limitations in this study. First, the small sample size increased the likelihood of missing other significant miRNA markers. Second, since our data were not enough to clarify whether the plasma miRNA profiles are either cause or effect, we cannot confirm the biological roles of these non-invasive markers in a clinical setting. Further miRNA profiling and their downstream gene analysis using human brain tissue from brain tissue bank can give a more direct answer. Brain tissue analysis with a gaming disorder animal model also would be helpful. Third, due to the limited availability of plasma samples, we examined only five downstream candidate molecules. Exploring more downstream targets with a larger sample set will be helpful to further understand the molecular mechanism of IGD.

In summary, through genome-wide screening of miRNA expression profiles and independent validation, we discovered three IGD-associated miRNAs (hsa-miR-200c-3p, hsa-miR-26b-5p, and hsa-miR-652-3p). Many of their downstream genes are reported to be involved in diverse neuropsychiatric disorders, and experimental validation of altered expression of these downstream genes support the implication of the miRNAs identified in this study. We found that individuals with downregulation of all three miRNA are at high risk of IGD. Together with the known clinical or environmental risk factors and diagnostic criteria, our findings can facilitate early intervention to help people at higher risk of IGD.

## Ethics Statement

This study was approved by the Institutional Review Board of the Catholic University Medical College of Korea (MC16SISI0120). All participants and their parents gave written informed consent.

## Author Contributions

ML and HC contributed equally to this paper. ML, D-JK, and Y-JC designed the study. SJ, S-MC, YP, DC, and JL performed experiments and data generation. J-WC, S-HP, J-SC, and D-JK collected blood samples and clinical information. ML, HC, S-HY, and Y-JC analyzed data. ML, HC, S-HY, and Y-JC described the manuscript. Y-JC supervised the project.

## Conflict of Interest Statement

The authors declare that the research was conducted in the absence of any commercial or financial relationships that could be construed as a potential conflict of interest.
