# Supplementary material for: Circulating MicroRNA Expression Levels Associated With Internet Gaming Disorder
Source: Front Psychiatry. 2018 Mar 12;9:81. doi: 10.3389/fpsyt.2018.00081 (PMC5858605; doi:10.3389/fpsyt.2018.00081)
Supplement: Supplementary file 1 [file data_sheet_1.docx]

Supplementary Material

Circulating microRNA expression levels associated with Internet Gaming Disorder

Minho Lee, Hyeyoung Cho, Seung Hyun Jung, Seon-Hee Yim, Sung-Min Cho, Ji-Won Chun, Soo-Hyun Paik, Yae Eun Park, Dong Huey Cheon, Ji Eun Lee, Jung-Seok Choi, Dai-Jin Kim, and Yeun-Jun Chung*

*** Correspondence:** Yeun-Jun Chung: [yejun@catholic.ac.kr](mailto:yejun@catholic.ac.kr)

**
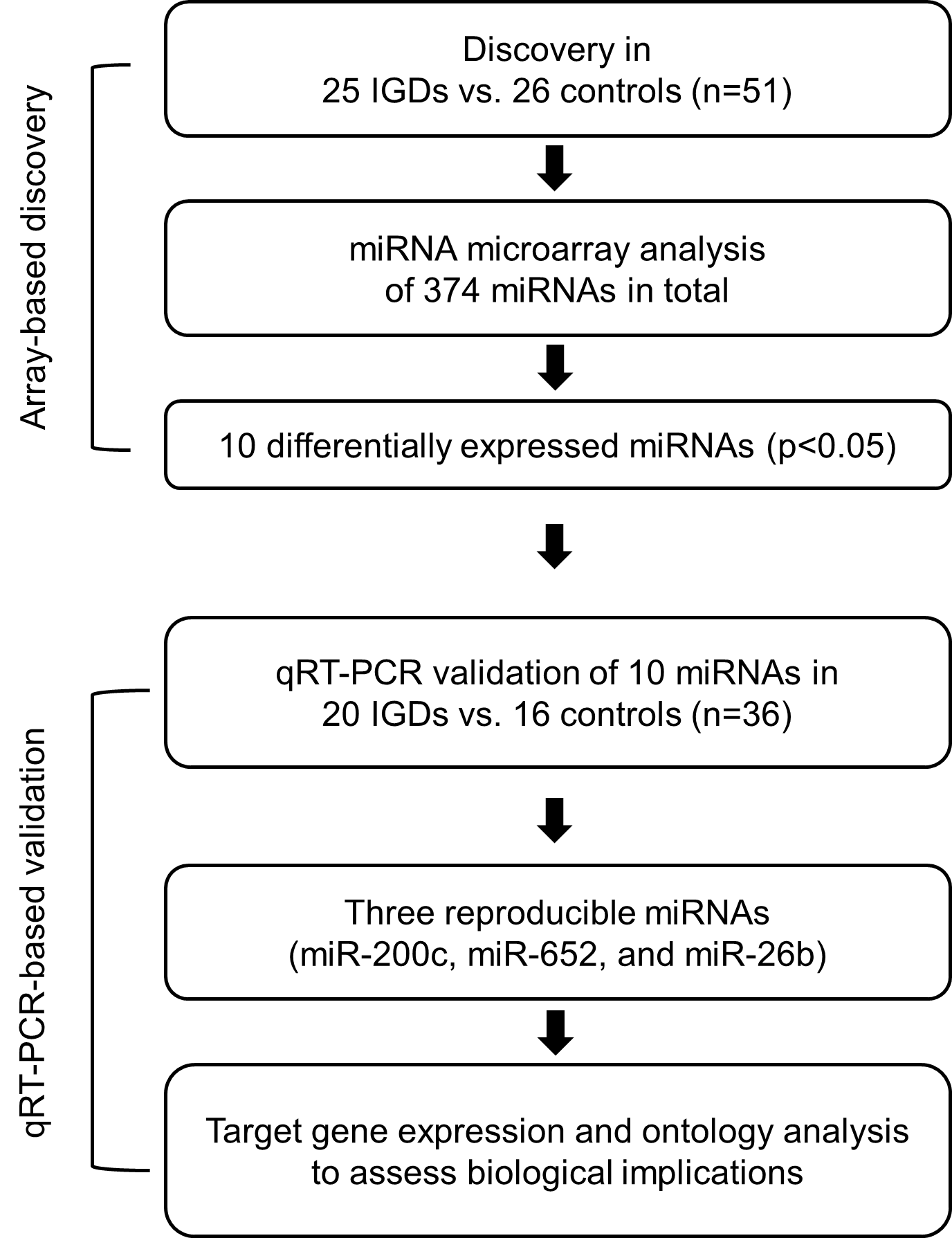
**

**Supplementary Figure 1. Study design and overall strategy for detecting miRNA markers of IGD**. To identify miRNA markers that can predict IGD, this study was conducted in two phases; (I) detection of differentially expressed (DE) miRNAs by miRNA array experiment; (II) validation of DE miRNAs by qRT-PCR and construction of the classifier

**Supplementary Table 1. miRNA expression levels measured by qRT-PCR.**

|  | ΔCt | | | | | | | | | |
| --- | --- | --- | --- | --- | --- | --- | --- | --- | --- | --- |
| Case | miR_29b_3p | miR_337c_5p | miR_411_5p | miR_200c_3p | miR_15b_5p | miR_125b_5p | miR_26b_5p | miR_652_3p | miR_483_5p | miR_423_5p |
| Control | 3.8783 | 5.3067 | 5.6630 | 4.2573 | -2.6077 | 2.9023 | -1.7387 | -0.2750 | 5.8507 | -1.8363 |
| Control | 3.9453 | 4.3620 | 4.6123 | 3.3880 | -3.1347 | 1.9360 | -2.2057 | 0.2290 | 4.9153 | -1.9010 |
| Control | 3.8697 | 3.8530 | 4.9210 | 3.2963 | -3.5180 | 1.7930 | -2.4350 | -0.5043 | 5.7063 | -1.6247 |
| Control | 4.4710 | 4.2930 | 4.9033 | 3.8800 | -2.7833 | 2.5153 | -1.9070 | -0.5547 | 7.2440 | -1.4513 |
| Control | 3.7610 | 3.5083 | 5.0947 | 3.9880 | -2.5697 | 2.1433 | -2.3430 | -0.9640 | 4.5803 | -2.5010 |
| Control | 4.2900 | 4.1897 | 5.0073 | 4.2833 | -2.9623 | 2.4167 | -2.0213 | 0.0063 | 5.1613 | -1.1860 |
| Control | 4.1893 | 4.3877 | 4.6177 | 3.4663 | -2.9903 | 2.8043 | -2.1603 | -0.6740 | 6.0613 | -1.3433 |
| Control | 4.5497 | 6.2790 | 5.8467 | 3.4223 | -2.8090 | 3.1320 | -1.6833 | -0.4730 | 7.3600 | -0.8070 |
| Control | 3.9567 | 4.3140 | 4.8867 | 2.8500 | -3.4700 | 2.2870 | -1.9973 | -1.1390 | 5.7933 | -1.6423 |
| Control | 4.6567 | 4.5217 | 4.1023 | 2.6443 | -3.2950 | 2.0143 | -1.9100 | -0.7483 | 4.4267 | -1.7657 |
| Control | 4.2323 | 3.4527 | 3.6483 | 3.0847 | -3.4357 | 2.0390 | -1.6877 | -0.8713 | 4.7480 | -1.6503 |
| Control | 4.5167 | 5.1437 | 6.2040 | 3.2300 | -3.2527 | 1.9640 | -1.9257 | -0.9070 | 5.5253 | -1.9733 |
| Control | 4.4620 | 4.5313 | 5.2077 | 3.7953 | -2.6483 | 2.6760 | -2.1737 | -0.7333 | 5.5203 | -1.3027 |
| Control | 3.6150 | 4.1613 | 4.7850 | 3.5727 | -3.2237 | 1.4443 | -2.7810 | -1.3193 | 2.1930 | -3.9603 |
| Control | 4.4803 | 4.3087 | 4.9480 | 3.9100 | -2.4870 | 2.1263 | -2.1320 | -0.6797 | 5.2557 | -1.8053 |
| Control | 4.4110 | 3.7707 | 4.3027 | 3.8050 | -2.8813 | 2.3137 | -1.2947 | -0.7953 | 4.0760 | -2.0757 |
| Control | 4.6457 | 4.1503 | 4.7233 | 3.7417 | -2.9187 | 2.9383 | -2.3467 | -0.3923 | 6.1383 | -1.1390 |
| Control | 4.3447 | 5.1587 | 5.4223 | 4.1137 | -2.6730 | 2.8963 | -1.3163 | -0.3117 | 5.7460 | -1.0323 |
| Control | 4.6613 | 5.8600 | 6.4973 | 4.0033 | -2.5317 | 2.8083 | -2.2303 | -0.5480 | 3.2487 | -1.5723 |
| Control | 5.2233 | 4.2810 | 4.5737 | 3.9843 | -2.5747 | 3.2530 | -2.2630 | -0.6610 | 5.0853 | -0.9590 |
| Control | 5.0577 | 4.5403 | 5.7867 | 4.2123 | -2.7847 | 2.6110 | -1.9537 | -0.4943 | 5.2490 | -1.1003 |
| Control | 5.0597 | 4.3913 | 5.2100 | 4.3303 | -2.2293 | 3.4520 | -0.7873 | -0.2373 | 5.8157 | -1.0360 |
| Control | 4.3600 | 2.9793 | 3.8737 | 4.0857 | -2.4410 | 3.0773 | -2.3953 | -0.5293 | 4.1060 | -1.9383 |
| Control | 4.1393 | 6.6060 | 7.7317 | 4.7130 | -2.7367 | 2.5370 | -2.0070 | -0.4323 | 3.4887 | -1.7613 |
| Control | 4.3037 | 3.5550 | 4.2810 | 4.3973 | -2.5820 | 3.3077 | -0.9463 | -0.3170 | 3.7550 | -2.0747 |
| Control | 3.1617 | 5.8894 | 5.8878 | 4.0063 | -3.1114 | 2.0462 | -2.1325 | -0.7643 | 2.4234 | -2.1764 |
| Control | 4.3923 | 5.4913 | 6.1410 | 4.4220 | -2.8180 | 2.6473 | -1.5980 | -0.7267 | 6.1123 | -1.3323 |
| IGD | 5.0350 | 4.5780 | 5.3450 | 4.3093 | -2.5353 | 2.9993 | -1.7070 | -0.1627 | 6.9687 | -1.1990 |
| IGD | 3.3210 | 4.4130 | 5.1007 | 2.9780 | -3.7443 | 2.1693 | -2.5837 | -0.4370 | 3.9337 | -2.0173 |
| IGD | 4.9703 | 3.8130 | 4.2857 | 4.8667 | -2.3197 | 3.2887 | -1.5473 | 0.0970 | 6.3127 | -1.2870 |
| IGD | 5.0163 | 4.3983 | 4.8870 | 4.6873 | -2.3950 | 3.1827 | -1.2803 | 0.0050 | 7.0163 | -0.6597 |
| IGD | 5.0013 | 3.7187 | 3.7510 | 4.0910 | -2.7480 | 3.0043 | -1.3610 | -0.0960 | 6.7973 | -0.7800 |
| IGD | 4.3287 | 3.9363 | 4.4720 | 3.8607 | -2.9783 | 2.3167 | -2.2117 | -0.5343 | 6.0157 | -1.3897 |
| IGD | 4.8840 | 5.2033 | 5.4237 | 3.6957 | -2.8683 | 2.1573 | -1.7203 | -0.4627 | 6.4457 | -1.2090 |
| IGD | 4.6153 | 6.0337 | 6.0347 | 4.4087 | -2.7697 | 2.7423 | -2.1523 | 0.0243 | 4.6683 | -1.4737 |
| IGD | 4.4567 | 4.7907 | 5.6157 | 4.0927 | -2.5500 | 3.0327 | -2.1123 | -0.2670 | 6.3940 | -1.0147 |
| IGD | 4.1080 | 4.1147 | 4.3060 | 3.7830 | -2.8343 | 2.3480 | -2.0950 | -0.4773 | 4.1357 | -1.7173 |
| IGD | 4.9417 | 4.4417 | 5.0203 | 4.3340 | -2.9097 | 2.3053 | -1.7837 | -0.1567 | 3.7853 | -2.1737 |
| IGD | 5.3090 | 4.6790 | 6.1420 | 4.6587 | -2.2730 | 2.6310 | -1.5590 | -0.0837 | 4.6183 | -1.1447 |
| IGD | 4.5710 | 3.7753 | 4.4587 | 4.4093 | -2.9010 | 2.7507 | -2.2323 | -0.3730 | 4.9603 | -1.7820 |
| IGD | 4.8650 | 4.6773 | 5.2333 | 7.0803 | -2.1133 | 3.5823 | -1.6017 | 0.3813 | 7.3757 | -0.4197 |
| IGD | 4.0463 | 5.6084 | 5.1570 | 4.3680 | -3.8466 | 1.7683 | -2.4099 | -1.7749 | 1.3237 | -3.8229 |
| IGD | 2.8777 | 7.1525 | 4.3550 | 5.0271 | -3.8099 | 0.9644 | -2.2581 | -0.8517 | 1.9761 | -3.5127 |
| IGD | 4.5301 | 5.9638 | 6.1444 | 4.1826 | -2.8835 | 3.0466 | -1.6954 | 0.0952 | 4.1459 | -0.7968 |
| IGD | 4.3979 | 4.9985 | 4.5248 | 3.7989 | -3.0497 | 2.7217 | -2.0036 | 0.0948 | 5.4771 | -0.9339 |
| IGD | 3.6006 | 3.6050 | 3.6341 | 3.0195 | -3.0050 | 2.5450 | -2.1164 | -0.1399 | 4.8490 | -1.4945 |
| IGD | 3.3665 | 5.0669 | 6.2150 | 3.8713 | -2.9972 | 2.4901 | -1.6566 | 0.0480 | 4.1516 | -1.6818 |
| IGD | 3.9666 | 5.0592 | 4.9973 | 3.7221 | -2.8474 | 2.7780 | -1.8728 | -0.0132 | 5.6743 | -1.0489 |
| IGD | 4.4686 | 5.0448 | 4.8552 | 4.2226 | -2.6610 | 2.8996 | -1.3522 | 0.2968 | 5.7283 | -0.6458 |
| IGD | 4.3429 | 5.5029 | 5.8846 | 4.2118 | -3.0045 | 2.8243 | -1.6851 | -0.1683 | 5.1290 | -0.8764 |
| IGD | 2.4324 | 3.0405 | 1.8529 | 4.6097 | -3.9818 | 1.2314 | -1.9592 | -1.4596 | 0.3209 | -3.7654 |
| IGD | 1.6518 | 0.9713 | 2.8788 | 0.0000 | -4.3859 | -0.2726 | -2.6898 | -2.6367 | 0.9019 | -4.9492 |
| IGD | 4.6268 | 5.5951 | 5.3433 | 4.4432 | -2.3189 | 2.8999 | -1.5577 | 0.0228 | 6.1912 | -0.4867 |
| IGD | 1.8632 | 2.3501 | 3.3299 | 11.4381 | -4.6982 | 0.4445 | -2.6424 | -2.1681 | -0.8117 | -4.5942 |
| IGD | 2.9788 | 4.1941 | 3.7096 | 4.9373 | -3.3562 | 2.0951 | -1.6050 | -1.4149 | 1.9813 | -2.3758 |

**Supplementary Table 2. The three miRNA candidate markers of IGD.** miR-26b-5p in the human database miRNAMap and miR-652-3p in a mouse model were reported to have detectable expressions in the brain, whereas miR-200c-3p was not.

| **miRNA** | **miRBase ID** | **Chromosomal** | **Location** | **Mature sequence** | **Expression levels** |
| --- | --- | --- | --- | --- | --- |
|  |  | **Region** |  |  | **in the brain** |
| hsa-miR-26b-5p | MIMAT0000083 | 2q35 | Intron | UUCAAGUAAUUCAGGAUAGGU | Moderate |
| hsa-miR-200c-3p | MIMAT0000617 | 12p13.31 | Intergenic | UAAUACUGCCGGGUAAUGAUGGA | None |
| hsa-miR-652-3p | MIMAT0003322 | Xq22.3 | Intron | AAUGGCGCCACUAGGGUUGUG | NA |
|  |  |  |  |  | (Moderate in mouse) |

**Supplementary Table 3. Relative Quantities of 3 miRNAs**

| Case | miR_26b_5p | miR_200c_3p | miR_652_3p |
| --- | --- | --- | --- |
| Control | 1.287195127 | 1.854609416 | 1.512527674 |
| Control | 1.150475905 | 1.596724471 | 0.733547116 |
| Control | 0.994629254 | 1.350145963 | 0.334704891 |
| Control | 1.238188332 | 1.153577863 | 1.977889103 |
| Control | 0.785253715 | 1.033913424 | 0.956582656 |
| Control | 1.066017239 | 0.956021776 | 1.598769038 |
| Control | 1.196011092 | 0.70374047 | 2.174918799 |
| Control | 0.96609085 | 1.080062938 | 0.927205827 |
| Control | 1.106674359 | 1.033197018 | 1.672448213 |
| Control | 0.796215449 | 0.922816295 | 0.580738884 |
| Control | 0.87310724 | 1.18930223 | 0.580336486 |
| Control | 1.838119764 | 1.079314555 | 0.679225034 |
| Control | 0.712139 | 0.27760691 | 0.472033625 |
| Control | 1.379581578 | 0.845053968 | 0.84907361 |
| Control | 0.7659015 | 2.318377317 | 0.714478022 |
| Control | 0.973485077 | 1.011934131 | 1.209123441 |
| Control | 1.160889592 | 1.132974495 | 0.997204666 |
| Control | 0.770694376 | 0.554444664 | 0.93236163 |
| Control | 0.972810543 | 1.068891476 | 1.441890431 |
| Control | 0.81463786 | 0.696944358 | 1.375505147 |
| Control | 0.892689752 | 0.943513709 | 0.903728633 |
| Control | 1.707908599 | 1.074835133 | 1.030940834 |
| Control | 0.882844202 | 0.940249399 | 1.471167525 |
| Control | 0.693626426 | 0.631176495 | 0.955919833 |
| Control | 1.030418763 | 1.263228289 | 1.369796461 |
| Control | 0.802309491 | 1.20423369 | 1.006230998 |
| Control | 1.053750224 | 1.189121959 | 0.546752772 |
| Control | 1.235304127 | 1.267128956 | 0.908963247 |
| Control | 0.85670254 | 0.845511738 | 0.941235214 |
| Control | 1.158988516 | 0.784527916 | 1.250028107 |
| Control | 1.021153398 | 1.126278299 | 1.022400562 |
| Control | 0.858485857 | 1.991096792 | 1.07645937 |
| Control | 0.735875158 | 1.467367281 | 1.172261041 |
| Control | 0.867859219 | 1.326750129 | 1.201603178 |
| Control | 1.030634605 | 0.89661675 | 1.065325181 |
| Control | 1.570110691 | 1.04625174 | 1.599137361 |
| Control | 1.001294438 | 0.828111358 | 1.026424279 |
| Control | 1.161937878 | 0.930598019 | 0.841067718 |
| Control | 0.568881316 | 0.719082907 | 0.79533094 |
| Control | 1.071921758 | 0.776233905 | 0.936895817 |
| Control | 1.201802314 | 0.733175266 | 0.924851645 |
| Control | 1.001622769 | 0.774614101 | 1.088437566 |
| IGD | 0.929309312 | 0.964675335 | 1.03739228 |
| IGD | 1.066756402 | 0.017278423 | 0.381819195 |
| IGD | 0.863477667 | 0.524173159 | 0.37839411 |
| IGD | 0.925452449 | 0.97003949 | 1.534705181 |
| IGD | 0.971462875 | 0.902572911 | 0.916344215 |
| IGD | 1.215231161 | 0.893856649 | 0.705130182 |
| IGD | 0.884681935 | 1.449058043 | 1.223455424 |
| IGD | 1.251129191 | 2.588498325 | 1.4200695 |
| IGD | 0.812945628 | 0.872432479 | 0.862718345 |
| IGD | 1.016232684 | 0.695978857 | 0.196412893 |
| IGD | 0.679351801 | 0.029119217 | 0.547511639 |
| IGD | 0.455092201 | 0.02812731 | 0.564076031 |
| IGD | 1.18692664 | 0.598372337 | 0.984840187 |
| IGD | 0.662150362 | 0.006293662 | 0.46392437 |
| IGD | 0.801198025 | 1.041826749 | 0.709542762 |
| IGD | 1.039025289 | 0.017776503 | 1.16874609 |
| IGD | 0.476395196 | 0.008717411 | 0.19505617 |
| IGD | 1.054991057 | 0.651625689 | 0.616409729 |
| IGD | 0.922890104 | 0.753727734 | 0.317309262 |
| IGD | 0.776593087 | 1.079314555 | 1.065079809 |
| IGD | 0.850999842 | 0.787370947 | 1.02737405 |
| IGD | 0.807889995 | 0.581203536 | 0.89500121 |
| IGD | 0.911447061 | 0.945477732 | 1.205775682 |
| IGD | 0.729121308 | 0.847987783 | 0.871130968 |
| IGD | 1.100554637 | 0.941553766 | 1.115709578 |
| IGD | 0.745802879 | 0.627881128 | 0.717289393 |
| IGD | 1.369389579 | 1.579969478 | 0.867514955 |
| IGD | 0.66766652 | 0.426681073 | 0.599138121 |
| IGD | 0.554861499 | 0.483156795 | 0.63858941 |
| IGD | 0.586769697 | 0.730469879 | 0.684897754 |
| IGD | 1.058141774 | 0.85691857 | 0.928062497 |
| IGD | 0.975941293 | 0.904314617 | 0.892110218 |
| IGD | 1.213974676 | 0.602862854 | 2.192940536 |
| IGD | 1.092746798 | 0.381776682 | 1.156380237 |
| IGD | 0.73985193 | 0.685522085 | 0.599885226 |
| IGD | 0.916035624 | 0.894384344 | 0.600052063 |
| IGD | 0.990502812 | 1.535195113 | 0.706069936 |
| IGD | 0.720177279 | 0.850623053 | 0.619848222 |
| IGD | 0.836605884 | 0.94329329 | 0.646685363 |
| IGD | 0.583218 | 0.666773968 | 0.521665222 |
| IGD | 0.73458782 | 0.671775595 | 0.720095685 |
| IGD | 0.88824053 | 0.509873558 | 1.762416933 |
| IGD | 0.67249318 | 0.572254703 | 0.630742849 |
| IGD | 1.426287784 | 0.004486527 | 2.879977843 |
| IGD | 0.694873957 | 0.40630377 | 1.708631503 |

**Supplementary Table 4. Target genes of the 3 miRNA candidate markers.**

1,230 genes were consistently predicted as downstream target gene by four algorithms (miRWalk, miRanda, RNA22, and Targetscan). 140 genes were common (target of >2 genes) target genes of two miRNAs

| Gene | hsa-miR-200c-3p | hsa-miR-26b-5p | hsa-miR-652-3p | Target of >2 genes |
| --- | --- | --- | --- | --- |
| ABAT | O |  |  |  |
| ABCA5 | O |  |  |  |
| ABI2 | O |  |  |  |
| ABL2 | O | O |  | O |
| ACADSB | O |  |  |  |
| ACVR2A | O |  |  |  |
| ADCY9 | O |  |  |  |
| AFF3 | O | O |  | O |
| AGAP1 | O |  |  |  |
| AGPS | O | O |  | O |
| AK4 | O |  |  |  |
| AKAP13 | O |  |  |  |
| ALDH7A1 | O |  |  |  |
| AMACR | O |  |  |  |
| AMER2 | O |  |  |  |
| AMER3 | O |  |  |  |
| AMFR | O |  |  |  |
| ANGEL2 | O |  |  |  |
| ANO5 | O |  |  |  |
| ANO6 | O |  |  |  |
| AP1AR | O |  |  |  |
| AP1S2 | O |  |  |  |
| APAF1 | O | O |  | O |
| APLP2 | O |  |  |  |
| AQP4 | O |  |  |  |
| ARHGEF26 | O |  |  |  |
| ARID4A | O |  |  |  |
| ARIH2 | O |  |  |  |
| ARL1 | O |  |  |  |
| ARL5A | O |  |  |  |
| ATF3 | O |  |  |  |
| ATL2 | O |  |  |  |
| ATP11C | O | O |  | O |
| ATP13A3 | O |  |  |  |
| ATP2A2 | O |  |  |  |
| ATP2B1 | O |  |  |  |
| ATP6V1E1 | O |  |  |  |
| ATRX | O | O |  | O |
| ATXN1 | O |  |  |  |
| B3GALTL | O |  |  |  |
| BAP1 | O |  |  |  |
| BBS12 | O |  |  |  |
| BCL2 | O |  |  |  |
| BDKRB2 | O |  |  |  |
| BMI1 | O |  |  |  |
| BMPER | O |  |  |  |
| BPTF | O |  |  |  |
| BRWD1 | O | O |  | O |
| BRWD3 | O |  |  |  |
| BTBD18 | O |  |  |  |
| BTBD9 | O |  |  |  |
| C11orf87 | O |  |  |  |
| C21orf91 | O |  |  |  |
| C22orf39 | O |  |  |  |
| C2orf42 | O |  |  |  |
| C5orf64 | O |  |  |  |
| C9orf171 | O |  |  |  |
| CA5B | O |  |  |  |
| CACNA2D1 | O |  |  |  |
| CACUL1 | O |  |  |  |
| CALCR | O |  |  |  |
| CALD1 | O |  |  |  |
| CALN1 | O |  |  |  |
| CAMSAP2 | O |  |  |  |
| CASP2 | O |  |  |  |
| CAST | O |  |  |  |
| CBL | O |  |  |  |
| CCDC144A | O |  |  |  |
| CCDC50 | O | O |  | O |
| CCNJ | O |  |  |  |
| CCNT2 | O |  |  |  |
| CCNYL1 | O |  |  |  |
| CCSAP | O |  |  |  |
| CD109 | O |  |  |  |
| CDC14B | O |  |  |  |
| CDC73 | O | O |  | O |
| CDCA4 | O |  |  |  |
| CDCA7 | O |  |  |  |
| CDH6 | O |  |  |  |
| CDHR1 | O |  |  |  |
| CDK14 | O |  |  |  |
| CDK16 | O |  |  |  |
| CDK17 | O |  |  |  |
| CDK6 | O | O |  | O |
| CDON | O |  |  |  |
| CDS2 | O |  |  |  |
| CELF1 | O | O |  | O |
| CEP350 | O | O |  | O |
| CEP41 | O |  |  |  |
| CEP85L | O |  |  |  |
| CERS6 | O | O |  | O |
| CFLAR | O | O |  | O |
| CHAC2 | O |  |  |  |
| CHD2 | O |  |  |  |
| CHM | O |  |  |  |
| CHML | O | O |  | O |
| CHRM2 | O |  |  |  |
| CHRNB2 | O | O |  | O |
| CHST11 | O |  | O | O |
| CLASP2 | O | O |  | O |
| CLDND1 | O |  |  |  |
| CLIP2 | O |  |  |  |
| CNKSR3 | O |  |  |  |
| CNOT6L | O | O |  | O |
| COG6 | O |  |  |  |
| COL4A3BP | O |  |  |  |
| COX11 | O |  |  |  |
| CPED1 | O | O |  | O |
| CPM | O |  | O | O |
| CPNE4 | O |  |  |  |
| CREBRF | O | O |  | O |
| CREG1 | O |  |  |  |
| CSMD3 | O |  |  |  |
| CSNK1G3 | O |  |  |  |
| CTSC | O |  |  |  |
| CUL5 | O |  |  |  |
| CYP1B1 | O |  |  |  |
| CYTH1 | O |  |  |  |
| CYTH3 | O |  |  |  |
| DCP2 | O |  |  |  |
| DCUN1D3 | O |  |  |  |
| DCX | O |  |  |  |
| DDIT4L | O |  |  |  |
| DDX3Y | O |  |  |  |
| DENND6A | O |  |  |  |
| DESI1 | O |  |  |  |
| DIRAS2 | O |  |  |  |
| DISC1 | O |  |  |  |
| DIXDC1 | O |  |  |  |
| DLC1 | O |  |  |  |
| DNA2 | O | O |  | O |
| DNAJB9 | O |  |  |  |
| DNAJC8 | O |  |  |  |
| DOCK4 | O | O |  | O |
| DPYSL2 | O |  | O | O |
| DRP2 | O |  |  |  |
| DSC3 | O |  |  |  |
| DTNA | O |  |  |  |
| DUSP1 | O |  |  |  |
| DUSP4 | O | O | O | O |
| EDNRA | O |  |  |  |
| EFCAB13 | O |  |  |  |
| EFNA1 | O |  |  |  |
| EGFR | O |  |  |  |
| EHD1 | O |  |  |  |
| EIF4B | O |  |  |  |
| EIF5 | O | O |  | O |
| EIF5A2 | O |  |  |  |
| ELF2 | O |  |  |  |
| ELK4 | O |  |  |  |
| ELL | O |  |  |  |
| ELL2 | O |  |  |  |
| EPDR1 | O |  |  |  |
| EPM2AIP1 | O | O |  | O |
| ERBB4 | O | O |  | O |
| ERG | O |  |  |  |
| ETV1 | O |  |  |  |
| ETV5 | O |  |  |  |
| EXOC5 | O |  |  |  |
| EXOSC3 | O |  |  |  |
| EXOSC9 | O |  |  |  |
| FAM105B | O | O |  | O |
| FAM169A | O |  |  |  |
| FAM179B | O |  |  |  |
| FAM210A | O |  |  |  |
| FAM218A | O |  |  |  |
| FAM46C | O | O |  | O |
| FAM60A | O |  |  |  |
| FAM76B | O |  |  |  |
| FAM84A | O | O |  | O |
| FAT3 | O |  |  |  |
| FEZ2 | O |  |  |  |
| FGD6 | O |  |  |  |
| FGF2 | O |  |  |  |
| FGF23 | O |  |  |  |
| FGF9 | O |  |  |  |
| FLI1 | O |  |  |  |
| FLT1 | O |  |  |  |
| FNDC3B | O |  |  |  |
| FOXN2 | O |  |  |  |
| FOXP1 | O |  |  |  |
| FOXP2 | O |  |  |  |
| FRAS1 | O |  |  |  |
| FRS2 | O |  |  |  |
| FZD4 | O |  |  |  |
| G2E3 | O | O |  | O |
| GATAD2B | O |  |  |  |
| GCNT2 | O |  |  |  |
| GDA | O |  |  |  |
| GDAP1 | O |  |  |  |
| GFPT1 | O | O |  | O |
| GIGYF1 | O |  |  |  |
| GIT2 | O |  |  |  |
| GJC1 | O |  | O | O |
| GMFB | O | O |  | O |
| GOLGA8A | O |  |  |  |
| GOLGA8B | O |  |  |  |
| GOSR2 | O |  |  |  |
| GPM6A | O |  |  |  |
| GPRIN3 | O |  |  |  |
| GRID2 | O |  |  |  |
| GRM5 | O |  |  |  |
| GSE1 | O | O |  | O |
| GSTCD | O |  |  |  |
| GTF2E1 | O |  |  |  |
| GXYLT1 | O |  |  |  |
| HAL | O |  |  |  |
| HDAC4 | O | O |  | O |
| HEG1 | O | O |  | O |
| HEMK1 | O |  |  |  |
| HIPK2 | O |  |  |  |
| HIPK3 | O |  |  |  |
| HK2 | O |  |  |  |
| HLCS | O |  |  |  |
| HLF | O |  |  |  |
| HMBOX1 | O |  |  |  |
| HMGCLL1 | O |  |  |  |
| HNF1B | O |  |  |  |
| HOOK1 | O |  |  |  |
| HPS5 | O |  |  |  |
| HS2ST1 | O |  |  |  |
| HS3ST3A1 | O |  |  |  |
| IDS | O |  |  |  |
| IFIT5 | O |  |  |  |
| IKZF2 | O |  |  |  |
| INTS7 | O | O |  | O |
| IPMK | O |  |  |  |
| ITGA10 | O | O |  | O |
| ITGA2 | O | O |  | O |
| ITPR1 | O |  |  |  |
| IYD | O |  |  |  |
| JAG2 | O |  |  |  |
| JAKMIP3 | O |  |  |  |
| JAZF1 | O |  |  |  |
| JHDM1D | O |  |  |  |
| JUN | O |  |  |  |
| KAL1 | O |  |  |  |
| KANK2 | O |  |  |  |
| KBTBD6 | O |  |  |  |
| KCNA6 | O |  |  |  |
| KCNC1 | O |  |  |  |
| KCTD12 | O |  |  |  |
| KCTD2 | O |  |  |  |
| KCTD8 | O |  |  |  |
| KDELC1 | O |  |  |  |
| KDM5A | O | O |  | O |
| KDR | O |  |  |  |
| KDSR | O |  |  |  |
| KIAA0430 | O |  |  |  |
| KIAA1244 | O |  |  |  |
| KIAA1432 | O |  |  |  |
| KIAA1456 | O |  |  |  |
| KIAA1462 | O |  |  |  |
| KIF24 | O |  |  |  |
| KLF6 | O |  |  |  |
| KLF9 | O |  |  |  |
| KLHDC4 | O |  |  |  |
| KLHL14 | O |  |  |  |
| KLHL3 | O |  |  |  |
| KLHL42 | O | O |  | O |
| KMT2E | O |  |  |  |
| KPNA6 | O | O |  | O |
| KRT80 | O |  |  |  |
| LANCL1 | O |  |  |  |
| LCOR | O |  |  |  |
| LCORL | O |  |  |  |
| LDLRAD4 | O |  |  |  |
| LEMD3 | O |  |  |  |
| LEPR | O |  |  |  |
| LMAN1 | O |  |  |  |
| LMBR1 | O | O |  | O |
| LONRF2 | O |  |  |  |
| LOX | O | O |  | O |
| LPAR5 | O |  |  |  |
| LPGAT1 | O |  |  |  |
| LPIN1 | O |  |  |  |
| LPPR4 | O |  |  |  |
| LRP1B | O |  |  |  |
| LRRC40 | O |  |  |  |
| LRRC58 | O |  |  |  |
| LTN1 | O | O |  | O |
| MACC1 | O |  |  |  |
| MALT1 | O | O |  | O |
| MAMDC2 | O |  |  |  |
| MAP1B | O | O |  | O |
| MAP2 | O | O |  | O |
| MAP3K1 | O |  |  |  |
| MAP3K13 | O | O |  | O |
| MAPRE1 | O |  |  |  |
| MARCH1 | O |  |  |  |
| MBNL3 | O | O |  | O |
| MCC | O |  |  |  |
| MCFD2 | O |  |  |  |
| MCMDC2 | O |  |  |  |
| MEF2D | O | O |  | O |
| MGAT5 | O |  |  |  |
| MIB1 | O |  |  |  |
| MIEF1 | O |  |  |  |
| MIER3 | O | O |  | O |
| MIPOL1 | O |  |  |  |
| MKI67 | O |  |  |  |
| MKLN1 | O | O |  | O |
| MLEC | O |  |  |  |
| MMAA | O |  |  |  |
| MMD2 | O |  |  |  |
| MOB1B | O | O |  | O |
| MON2 | O |  |  |  |
| MORC3 | O |  |  |  |
| MPP5 | O |  |  |  |
| MRVI1 | O |  |  |  |
| MSL2 | O |  |  |  |
| MSN | O |  |  |  |
| MTAP | O |  |  |  |
| MTMR9 | O |  |  |  |
| MUL1 | O |  |  |  |
| MYCT1 | O |  |  |  |
| MYO5A | O |  |  |  |
| MYO9A | O | O |  | O |
| NAA16 | O |  |  |  |
| NAB1 | O | O |  | O |
| NALCN | O |  |  |  |
| NAMPT | O |  |  |  |
| NANOS1 | O |  |  |  |
| NAP1L1 | O |  |  |  |
| NAP1L5 | O |  |  |  |
| NBR1 | O |  |  |  |
| NCEH1 | O | O |  | O |
| NCK1 | O |  |  |  |
| NCOA3 | O |  |  |  |
| NCOA4 | O | O |  | O |
| NCR3LG1 | O |  |  |  |
| NEO1 | O |  |  |  |
| NFASC | O |  |  |  |
| NFIB | O |  |  |  |
| NIPA1 | O | O |  | O |
| NLGN4Y | O |  |  |  |
| NOTCH1 | O |  |  |  |
| NOVA1 | O |  |  |  |
| NPAP1 | O |  |  |  |
| NPNT | O |  |  |  |
| NPTX1 | O |  |  |  |
| NRBF2 | O |  |  |  |
| NRIP1 | O | O |  | O |
| NRIP3 | O | O |  | O |
| NRXN1 | O | O |  | O |
| NTF3 | O |  |  |  |
| NTRK2 | O | O |  | O |
| NUFIP2 | O | O |  | O |
| NUMB | O |  |  |  |
| OLFML2A | O |  |  |  |
| ONECUT2 | O |  | O | O |
| OSBP | O |  |  |  |
| OSBPL11 | O |  |  |  |
| OSBPL6 | O |  |  |  |
| OSGIN2 | O | O |  | O |
| OSTM1 | O |  |  |  |
| OTUD6B | O | O |  | O |
| OTUD7B | O |  |  |  |
| OXR1 | O |  |  |  |
| PAIP2 | O |  |  |  |
| PAK2 | O |  |  |  |
| PAQR5 | O |  |  |  |
| PARD3 | O |  |  |  |
| PARD3B | O |  |  |  |
| PBX3 | O |  |  |  |
| PCDH11X | O |  |  |  |
| PCNX | O |  |  |  |
| PDCD10 | O |  |  |  |
| PDE10A | O |  |  |  |
| PDHA1 | O |  |  |  |
| PDPR | O |  |  |  |
| PDS5B | O |  |  |  |
| PEAK1 | O |  |  |  |
| PELI2 | O | O |  | O |
| PEX2 | O |  |  |  |
| PHEX | O |  |  |  |
| PHF20L1 | O |  |  |  |
| PHF21B | O |  |  |  |
| PHKB | O |  |  |  |
| PI15 | O | O | O | O |
| PI4KB | O |  |  |  |
| PIGA | O |  |  |  |
| PIGM | O |  |  |  |
| PIKFYVE | O | O |  | O |
| PIM2 | O |  |  |  |
| PIP4K2A | O |  |  |  |
| PIP4K2B | O | O |  | O |
| PITPNA | O |  |  |  |
| PITPNM3 | O |  |  |  |
| PKD2 | O |  |  |  |
| PKIA | O |  |  |  |
| PKP1 | O |  |  |  |
| PLAA | O |  |  |  |
| PLAG1 | O |  | O | O |
| PLCG1 | O |  |  |  |
| PLXNA4 | O |  |  |  |
| PMM1 | O |  |  |  |
| PPARA | O |  |  |  |
| PPFIA1 | O |  |  |  |
| PPFIBP1 | O |  |  |  |
| PPM1H | O | O |  | O |
| PPP1CB | O |  |  |  |
| PPP1R12B | O | O |  | O |
| PPP1R18 | O |  |  |  |
| PPP1R9A | O | O |  | O |
| PPP2R1B | O |  |  |  |
| PPP6C | O |  |  |  |
| PPP6R3 | O |  |  |  |
| PRDM15 | O |  |  |  |
| PRKAB1 | O |  |  |  |
| PRKACB | O |  |  |  |
| PRKCH | O |  |  |  |
| PRKG1 | O |  |  |  |
| PRLR | O | O |  | O |
| PROK2 | O |  |  |  |
| PRTG | O | O |  | O |
| PTBP1 | O |  |  |  |
| PTGER3 | O | O |  | O |
| PTGFR | O |  |  |  |
| PTP4A1 | O | O |  | O |
| PTPN11 | O |  |  |  |
| PTPN21 | O |  |  |  |
| PTPN22 | O |  |  |  |
| PVRL1 | O |  |  |  |
| PVRL4 | O |  |  |  |
| QKI | O |  |  |  |
| RAB11FIP2 | O |  |  |  |
| RAB18 | O | O |  | O |
| RAB27B | O |  |  |  |
| RAB3B | O |  |  |  |
| RAB8B | O |  |  |  |
| RAD18 | O |  |  |  |
| RAG1 | O |  |  |  |
| RALGPS2 | O |  |  |  |
| RANBP6 | O |  |  |  |
| RAP1B | O |  |  |  |
| RAP2B | O |  |  |  |
| RAPGEF2 | O |  |  |  |
| RASA2 | O | O |  | O |
| RASSF6 | O |  |  |  |
| RASSF8 | O |  |  |  |
| RBL1 | O |  |  |  |
| RBM20 | O | O |  | O |
| RBM46 | O | O |  | O |
| RDH10 | O |  |  |  |
| RDX | O |  |  |  |
| REEP3 | O | O |  | O |
| RELN | O |  |  |  |
| REV3L | O |  |  |  |
| RFX7 | O |  |  |  |
| RHOBTB1 | O |  |  |  |
| RHOT1 | O |  |  |  |
| RIF1 | O |  |  |  |
| RIMKLB | O |  |  |  |
| RIPK2 | O |  |  |  |
| RIPK4 | O |  |  |  |
| RLF | O |  |  |  |
| RND3 | O |  |  |  |
| RNF180 | O |  |  |  |
| RNF2 | O |  |  |  |
| RNF213 | O |  |  |  |
| RNF39 | O |  |  |  |
| RNGTT | O |  |  |  |
| ROBO2 | O |  |  |  |
| RORB | O |  |  |  |
| RPE | O |  |  |  |
| RPL22L1 | O |  |  |  |
| RPL28 | O |  | O | O |
| RPRD1A | O |  |  |  |
| RPS6KA3 | O |  |  |  |
| RPS6KB1 | O |  |  |  |
| RRP15 | O |  |  |  |
| RSF1 | O |  |  |  |
| RSU1 | O |  |  |  |
| RTF1 | O | O |  | O |
| RTKN2 | O |  |  |  |
| RUSC2 | O |  |  |  |
| S100PBP | O |  |  |  |
| SASH1 | O |  |  |  |
| SBNO1 | O |  |  |  |
| SCD | O |  |  |  |
| SCML1 | O |  |  |  |
| SCN2A | O |  |  |  |
| SCN3A | O |  |  |  |
| SCN9A | O | O |  | O |
| SCO1 | O |  |  |  |
| SCOC | O |  |  |  |
| SCP2 | O |  |  |  |
| SCRT2 | O |  |  |  |
| SDC2 | O |  |  |  |
| SEC23A | O |  |  |  |
| SFTPA1 | O |  |  |  |
| SGCD | O |  |  |  |
| SGPP1 | O |  |  |  |
| SGTB | O |  |  |  |
| SH2D1B | O |  |  |  |
| SH3BP2 | O | O |  | O |
| SH3PXD2A | O | O |  | O |
| SH3RF2 | O |  |  |  |
| SHC4 | O |  |  |  |
| SHE | O |  |  |  |
| SHOC2 | O |  |  |  |
| SHROOM4 | O | O |  | O |
| SIAH1 | O |  |  |  |
| SIKE1 | O |  |  |  |
| SKA2 | O |  |  |  |
| SLC12A2 | O | O |  | O |
| SLC15A2 | O |  |  |  |
| SLC1A2 | O |  |  |  |
| SLC25A27 | O |  |  |  |
| SLC25A30 | O |  |  |  |
| SLC26A2 | O | O |  | O |
| SLC30A7 | O | O |  | O |
| SLC35B4 | O | O |  | O |
| SLC35D1 | O |  |  |  |
| SLC35F4 | O |  |  |  |
| SLC38A2 | O | O |  | O |
| SLC39A9 | O |  |  |  |
| SLC4A7 | O | O |  | O |
| SLC5A3 | O | O |  | O |
| SLC6A4 | O |  |  |  |
| SLIT1 | O |  | O | O |
| SLIT2 | O |  |  |  |
| SMAD2 | O | O |  | O |
| SMAD7 | O |  |  |  |
| SMARCA5 | O |  |  |  |
| SMARCD1 | O |  |  |  |
| SMC1A | O |  |  |  |
| SNAP25 | O |  |  |  |
| SNAPC1 | O |  |  |  |
| SNTB2 | O |  |  |  |
| SNX1 | O |  |  |  |
| SNX16 | O |  |  |  |
| SOAT1 | O |  |  |  |
| SOCS4 | O |  |  |  |
| SOCS6 | O | O |  | O |
| SOGA3 | O | O |  | O |
| SOS1 | O |  |  |  |
| SOWAHC | O |  |  |  |
| SP1 | O |  |  |  |
| SPAST | O |  |  |  |
| SPTBN1 | O | O |  | O |
| SPTLC3 | O |  |  |  |
| SRGAP3 | O |  |  |  |
| SRSF2 | O |  |  |  |
| SSFA2 | O |  |  |  |
| SSR1 | O | O |  | O |
| ST3GAL2 | O |  |  |  |
| ST6GAL2 | O |  |  |  |
| ST7 | O |  |  |  |
| ST8SIA3 | O | O |  | O |
| STK10 | O |  |  |  |
| STK25 | O |  |  |  |
| STK38L | O |  |  |  |
| STX16 | O |  |  |  |
| STYX | O |  |  |  |
| SUSD5 | O |  |  |  |
| SWAP70 | O |  |  |  |
| SYDE1 | O |  |  |  |
| SYVN1 | O |  |  |  |
| TACC1 | O |  |  |  |
| TAF12 | O | O |  | O |
| TAP2 | O |  |  |  |
| TBC1D15 | O | O |  | O |
| TBP | O |  |  |  |
| TBX18 | O |  |  |  |
| TBX22 | O |  |  |  |
| TCF4 | O |  |  |  |
| TCP11L2 | O |  |  |  |
| TENM1 | O |  |  |  |
| TET2 | O | O |  | O |
| TFE3 | O |  |  |  |
| TGFBR1 | O |  |  |  |
| THAP2 | O | O |  | O |
| THRB | O | O |  | O |
| TIMP2 | O |  |  |  |
| TLL1 | O |  |  |  |
| TM2D2 | O |  |  |  |
| TMA16 | O |  |  |  |
| TMEM136 | O |  |  |  |
| TMEM164 | O |  |  |  |
| TMEM167A | O |  |  |  |
| TMEM170B | O |  |  |  |
| TMEM237 | O |  |  |  |
| TMEM25 | O |  |  |  |
| TMOD3 | O |  |  |  |
| TNFAIP1 | O |  |  |  |
| TNNI1 | O |  |  |  |
| TOR1AIP2 | O |  |  |  |
| TP53INP1 | O |  |  |  |
| TP73 | O |  |  |  |
| TRANK1 | O |  |  |  |
| TRAPPC10 | O |  |  |  |
| TRAPPC6B | O |  |  |  |
| TRIM23 | O |  |  |  |
| TRIML1 | O |  |  |  |
| TRIO | O |  |  |  |
| TROVE2 | O | O |  | O |
| TSC1 | O |  |  |  |
| TSC22D2 | O |  |  |  |
| TSSK1B | O |  |  |  |
| TTC3 | O |  |  |  |
| TULP3 | O |  |  |  |
| TULP4 | O |  |  |  |
| TXLNG | O |  |  |  |
| TYW5 | O |  |  |  |
| UBA6 | O |  |  |  |
| UBE2B | O |  |  |  |
| UBE2W | O | O |  | O |
| UBN2 | O | O |  | O |
| UNC80 | O |  |  |  |
| UPK1B | O |  |  |  |
| USH1G | O |  |  |  |
| USH2A | O | O |  | O |
| USP15 | O | O |  | O |
| USP18 | O |  |  |  |
| USP25 | O | O |  | O |
| USP31 | O |  |  |  |
| USP47 | O |  |  |  |
| VAMP4 | O |  |  |  |
| VANGL2 | O |  |  |  |
| VAT1L | O |  |  |  |
| VGLL3 | O | O |  | O |
| VLDLR | O |  |  |  |
| WAPAL | O |  |  |  |
| WASF3 | O |  |  |  |
| WDR82 | O |  |  |  |
| WDR91 | O |  |  |  |
| WHSC1 | O |  |  |  |
| WIF1 | O |  |  |  |
| WNT5A | O | O |  | O |
| XPO4 | O | O |  | O |
| YAF2 | O |  |  |  |
| YWHAB | O |  |  |  |
| YWHAG | O |  |  |  |
| ZBTB44 | O |  |  |  |
| ZBTB5 | O |  |  |  |
| ZBTB7C | O |  |  |  |
| ZC3H12B | O |  |  |  |
| ZC3H13 | O |  |  |  |
| ZC3H4 | O |  |  |  |
| ZC3H6 | O |  |  |  |
| ZCCHC14 | O |  |  |  |
| ZCCHC24 | O | O |  | O |
| ZEB1 | O |  |  |  |
| ZFAND5 | O |  | O | O |
| ZFX | O | O |  | O |
| ZFYVE20 | O |  |  |  |
| ZIC3 | O |  |  |  |
| ZMAT3 | O |  |  |  |
| ZNF148 | O | O |  | O |
| ZNF181 | O |  |  |  |
| ZNF33A | O |  |  |  |
| ZNF451 | O | O |  | O |
| ZNF516 | O |  |  |  |
| ZNF529 | O |  |  |  |
| ZNF555 | O | O |  | O |
| ZNF557 | O |  |  |  |
| ZNF566 | O |  |  |  |
| ZNF605 | O |  |  |  |
| ZNF641 | O |  |  |  |
| ZNF662 | O | O |  | O |
| ZNF674 | O |  |  |  |
| ZNF704 | O | O |  | O |
| ZNHIT6 | O | O |  | O |
| ZSWIM4 | O |  |  |  |
| ZXDA | O |  |  |  |
| ZYG11B | O |  |  |  |
| ABCD4 |  | O |  |  |
| ABCE1 |  | O |  |  |
| ABHD2 |  | O |  |  |
| ACAP2 |  | O |  |  |
| ACBD5 |  | O |  |  |
| ACKR4 |  | O |  |  |
| ACSL4 |  | O |  |  |
| ACSS1 |  | O |  |  |
| ACTN2 |  | O |  |  |
| ADAM19 |  | O |  |  |
| ADAM9 |  | O |  |  |
| ADAMTS19 |  | O |  |  |
| ADAMTS6 |  | O |  |  |
| AMMECR1 |  | O |  |  |
| ANKRD28 |  | O |  |  |
| ANKRD52 |  | O |  |  |
| ANKS1A |  | O |  |  |
| APC |  | O |  |  |
| ARAP2 |  | O |  |  |
| ARHGAP26 |  | O |  |  |
| ARL4A |  | O |  |  |
| ARL4C |  | O |  |  |
| ARPC3 |  | O |  |  |
| ARPP19 |  | O |  |  |
| ARSB |  | O |  |  |
| ASCC3 |  | O |  |  |
| ASPN |  | O |  |  |
| ASRGL1 |  | O |  |  |
| ATAD2B |  | O |  |  |
| ATF2 |  | O |  |  |
| ATP1A2 |  | O |  |  |
| ATPAF1 |  | O |  |  |
| ATXN3 |  | O |  |  |
| B3GNT5 |  | O |  |  |
| B4GALT4 |  | O |  |  |
| BAK1 |  | O |  |  |
| BAZ2B |  | O |  |  |
| BBX |  | O |  |  |
| BCAT1 |  | O |  |  |
| BCL7B |  | O |  |  |
| BCR |  | O |  |  |
| BDP1 |  | O |  |  |
| BMPR1B |  | O |  |  |
| C12orf23 |  | O |  |  |
| C12orf49 |  | O |  |  |
| C14orf105 |  | O |  |  |
| C19orf66 |  | O |  |  |
| C1GALT1 |  | O |  |  |
| C1orf27 |  | O |  |  |
| C1orf53 |  | O |  |  |
| C2CD2 |  | O |  |  |
| C3orf58 |  | O |  |  |
| C5orf22 |  | O |  |  |
| C5orf24 |  | O |  |  |
| C9orf40 |  | O |  |  |
| CACNG8 |  | O |  |  |
| CALCRL |  | O |  |  |
| CAMSAP1 |  | O |  |  |
| CASZ1 |  | O |  |  |
| CBLB |  | O |  |  |
| CCDC28A |  | O |  |  |
| CCDC6 |  | O |  |  |
| CCNJL |  | O |  |  |
| CD200 |  | O |  |  |
| CD93 |  | O |  |  |
| CDV3 |  | O |  |  |
| CECR2 |  | O |  |  |
| CELF2 |  | O |  |  |
| CHCHD7 |  | O |  |  |
| CHST3 |  | O | O | O |
| CLIC5 |  | O |  |  |
| CNOT4 |  | O |  |  |
| CNPY1 |  | O |  |  |
| CNR1 |  | O | O | O |
| CNTN5 |  | O |  |  |
| COL10A1 |  | O |  |  |
| CPNE3 |  | O |  |  |
| CPSF2 |  | O |  |  |
| CTBS |  | O |  |  |
| CTTNBP2NL |  | O |  |  |
| CXADR |  | O |  |  |
| DAPK1 |  | O |  |  |
| DBF4B |  | O |  |  |
| DCAF10 |  | O |  |  |
| DCAF4L2 |  | O |  |  |
| DCBLD2 |  | O |  |  |
| DDHD2 |  | O |  |  |
| DDX52 |  | O |  |  |
| DENND1B |  | O |  |  |
| DENND5B |  | O |  |  |
| DEPDC1 |  | O |  |  |
| DFFB |  | O |  |  |
| DGCR14 |  | O |  |  |
| DGKB |  | O |  |  |
| DLG5 |  | O |  |  |
| DMD |  | O |  |  |
| DNAJB5 |  | O |  |  |
| DNMBP |  | O |  |  |
| DNPEP |  | O |  |  |
| DTD2 |  | O |  |  |
| E2F7 |  | O |  |  |
| ECHDC2 |  | O |  |  |
| EDEM3 |  | O |  |  |
| EFR3A |  | O |  |  |
| EFTUD2 |  | O |  |  |
| EIF2S1 |  | O |  |  |
| EIF3A |  | O |  |  |
| ENC1 |  | O |  |  |
| EPB41L5 |  | O |  |  |
| EPC1 |  | O |  |  |
| EPC2 |  | O |  |  |
| EPHA2 |  | O |  |  |
| EPHA5 |  | O |  |  |
| EPS15 |  | O |  |  |
| EPT1 |  | O |  |  |
| ERC1 |  | O |  |  |
| ERC2 |  | O |  |  |
| ERICH2 |  | O |  |  |
| ESR1 |  | O |  |  |
| EZH2 |  | O |  |  |
| FAM136A |  | O |  |  |
| FAM160A1 |  | O |  |  |
| FAM172A |  | O |  |  |
| FAM199X |  | O |  |  |
| FAM216B |  | O |  |  |
| FAM222B |  | O |  |  |
| FAM8A1 |  | O |  |  |
| FAM92A1 |  | O |  |  |
| FBXL19 |  | O |  |  |
| FBXO11 |  | O |  |  |
| FCAR |  | O |  |  |
| FGFR1OP2 |  | O |  |  |
| FGL2 |  | O |  |  |
| FKTN |  | O |  |  |
| FLVCR1 |  | O |  |  |
| FMO5 |  | O |  |  |
| FNIP1 |  | O |  |  |
| FOXJ3 |  | O |  |  |
| FOXN3 |  | O |  |  |
| FOXO1 |  | O |  |  |
| FRZB |  | O |  |  |
| FSTL5 |  | O |  |  |
| G3BP2 |  | O |  |  |
| GABRA4 |  | O |  |  |
| GABRB2 |  | O | O | O |
| GABRG1 |  | O |  |  |
| GALNT10 |  | O |  |  |
| GBP1 |  | O |  |  |
| GFRA1 |  | O |  |  |
| GGA2 |  | O |  |  |
| GJA3 |  | O |  |  |
| GNPNAT1 |  | O |  |  |
| GON4L |  | O |  |  |
| GPR12 |  | O |  |  |
| GPR155 |  | O |  |  |
| GRAMD1C |  | O |  |  |
| GRB10 |  | O |  |  |
| GREB1 |  | O |  |  |
| GRHL3 |  | O |  |  |
| GRIN2A |  | O |  |  |
| GRIN3A |  | O |  |  |
| GRSF1 |  | O |  |  |
| GSK3B |  | O |  |  |
| GTF2A2 |  | O |  |  |
| GYPE |  | O |  |  |
| HAPLN1 |  | O |  |  |
| HAS3 |  | O |  |  |
| HCN3 |  | O |  |  |
| HECTD3 |  | O |  |  |
| HELZ |  | O |  |  |
| HEPHL1 |  | O |  |  |
| HGF |  | O |  |  |
| HMGA1 |  | O |  |  |
| HMGA2 |  | O |  |  |
| HMGB3 |  | O |  |  |
| HOXA5 |  | O |  |  |
| HOXA9 |  | O |  |  |
| HPGD |  | O |  |  |
| HPSE |  | O |  |  |
| HSD17B6 |  | O |  |  |
| HSPA12A |  | O |  |  |
| HSPA13 |  | O |  |  |
| HSPA14 |  | O |  |  |
| IARS |  | O |  |  |
| IL18R1 |  | O |  |  |
| INHBB |  | O |  |  |
| INTS2 |  | O |  |  |
| IQCJ |  | O |  |  |
| IRF4 |  | O |  |  |
| ITGA5 |  | O |  |  |
| ITPRIP |  | O |  |  |
| IVD |  | O | O | O |
| JAG1 |  | O |  |  |
| JAM2 |  | O |  |  |
| KCNA3 |  | O |  |  |
| KCNJ2 |  | O |  |  |
| KCTD20 |  | O |  |  |
| KIAA1024 |  | O |  |  |
| KIAA1328 |  | O |  |  |
| KIAA1468 |  | O |  |  |
| KIAA1549L |  | O |  |  |
| KIAA1958 |  | O |  |  |
| KIAA2013 |  | O |  |  |
| KIAA2022 |  | O |  |  |
| KIF5C |  | O |  |  |
| KLF11 |  | O |  |  |
| KLF12 |  | O |  |  |
| KLF7 |  | O |  |  |
| KPNA2 |  | O |  |  |
| LAMA1 |  | O |  |  |
| LARP4 |  | O |  |  |
| LETM1 |  | O |  |  |
| LGR4 |  | O |  |  |
| LIN28B |  | O |  |  |
| LMLN |  | O |  |  |
| LPP |  | O |  |  |
| LRRC2 |  | O |  |  |
| LRRC55 |  | O |  |  |
| LSM2 |  | O |  |  |
| MAB21L1 |  | O |  |  |
| MAP10 |  | O |  |  |
| MAP3K2 |  | O |  |  |
| MAPK6 |  | O |  |  |
| MAPRE2 |  | O |  |  |
| MAT2A |  | O |  |  |
| MCTP2 |  | O |  |  |
| MDM4 |  | O | O | O |
| MECP2 |  | O |  |  |
| METAP2 |  | O |  |  |
| METTL15 |  | O |  |  |
| MEX3B |  | O |  |  |
| MFHAS1 |  | O |  |  |
| MGAT4A |  | O |  |  |
| MME |  | O |  |  |
| MPP7 |  | O |  |  |
| MRAS |  | O |  |  |
| MRPL1 |  | O |  |  |
| MSMO1 |  | O |  |  |
| MTDH |  | O |  |  |
| MTERFD2 |  | O |  |  |
| MTM1 |  | O |  |  |
| MTMR12 |  | O |  |  |
| MTTP |  | O |  |  |
| MXI1 |  | O |  |  |
| MYH10 |  | O |  |  |
| MYLK4 |  | O |  |  |
| MYNN |  | O |  |  |
| NACC2 |  | O |  |  |
| NDUFA4 |  | O |  |  |
| NEBL |  | O |  |  |
| NEK4 |  | O |  |  |
| NFATC2IP |  | O |  |  |
| NFE2L3 |  | O |  |  |
| NHS |  | O |  |  |
| NIPAL4 |  | O |  |  |
| NOS1 |  | O |  |  |
| NT5DC1 |  | O |  |  |
| NTN4 |  | O |  |  |
| NUCKS1 |  | O |  |  |
| NUDT11 |  | O |  |  |
| NUDT21 |  | O |  |  |
| NUP50 |  | O |  |  |
| NUP54 |  | O |  |  |
| NUS1 |  | O |  |  |
| OPCML |  | O |  |  |
| OSBPL2 |  | O |  |  |
| OTUD3 |  | O |  |  |
| OXNAD1 |  | O |  |  |
| PAFAH1B2 |  | O |  |  |
| PAK1 |  | O |  |  |
| PALM2 |  | O |  |  |
| PAPD4 |  | O |  |  |
| PAPPA |  | O |  |  |
| PAQR8 |  | O |  |  |
| PAX2 |  | O |  |  |
| PCCB |  | O | O | O |
| PCDH18 |  | O |  |  |
| PCDH9 |  | O |  |  |
| PCK1 |  | O |  |  |
| PCSK5 |  | O |  |  |
| PDCD6IP |  | O |  |  |
| PDE4C |  | O |  |  |
| PDE4D |  | O |  |  |
| PDHX |  | O |  |  |
| PDSS2 |  | O |  |  |
| PEG10 |  | O |  |  |
| PFKFB2 |  | O |  |  |
| PFKFB3 |  | O |  |  |
| PGM2 |  | O |  |  |
| PGR |  | O |  |  |
| PHF6 |  | O |  |  |
| PHLDB2 |  | O |  |  |
| PHYHIPL |  | O |  |  |
| PLCXD3 |  | O |  |  |
| PLEKHG1 |  | O |  |  |
| PLXNA2 |  | O |  |  |
| POLR3G |  | O |  |  |
| PPM1B |  | O |  |  |
| PPP1R3A |  | O |  |  |
| PPP1R3D |  | O |  |  |
| PPP2R3C |  | O |  |  |
| PPP2R5C |  | O |  |  |
| PRDX3 |  | O |  |  |
| PREPL |  | O |  |  |
| PRKAA1 |  | O |  |  |
| PRKAA2 |  | O |  |  |
| PRKCD |  | O |  |  |
| PRPF40A |  | O |  |  |
| PSD3 |  | O |  |  |
| PTBP3 |  | O |  |  |
| PTGS2 |  | O |  |  |
| PTPN4 |  | O |  |  |
| PTPRB |  | O |  |  |
| PTPRD |  | O |  |  |
| PURB |  | O | O | O |
| RAB11A |  | O |  |  |
| RAB22A |  | O |  |  |
| RAB31 |  | O |  |  |
| RAB39B |  | O |  |  |
| RAB3IP |  | O |  |  |
| RAD1 |  | O |  |  |
| RAP2C |  | O |  |  |
| RASSF3 |  | O |  |  |
| RB1 |  | O |  |  |
| RBBP9 |  | O |  |  |
| RBM24 |  | O |  |  |
| RBMS3 |  | O |  |  |
| RCOR1 |  | O |  |  |
| RFX3 |  | O |  |  |
| RGS4 |  | O |  |  |
| RIOK3 |  | O |  |  |
| RNMT |  | O |  |  |
| RPS6KA2 |  | O |  |  |
| RRAGD |  | O |  |  |
| RUFY3 |  | O |  |  |
| S100A7A |  | O |  |  |
| SAMD8 |  | O |  |  |
| SCAPER |  | O |  |  |
| SDE2 |  | O |  |  |
| SEC24A |  | O |  |  |
| SELP |  | O |  |  |
| SEMA4D |  | O |  |  |
| SERBP1 |  | O | O | O |
| SESTD1 |  | O |  |  |
| SETBP1 |  | O |  |  |
| SETDB2 |  | O |  |  |
| SFPQ |  | O |  |  |
| SFRP4 |  | O |  |  |
| SFT2D3 |  | O |  |  |
| SGCB |  | O |  |  |
| SGMS2 |  | O |  |  |
| SH3KBP1 |  | O |  |  |
| SLAIN2 |  | O |  |  |
| SLBP |  | O |  |  |
| SLC16A6 |  | O |  |  |
| SLC19A2 |  | O |  |  |
| SLC1A4 |  | O |  |  |
| SLC22A23 |  | O |  |  |
| SLC22A5 |  | O |  |  |
| SLC2A13 |  | O |  |  |
| SLC2A14 |  | O |  |  |
| SLC2A3 |  | O |  |  |
| SLC2A4 |  | O |  |  |
| SLC4A4 |  | O |  |  |
| SLC7A1 |  | O | O | O |
| SLC7A6 |  | O |  |  |
| SLC9A8 |  | O |  |  |
| SMAD4 |  | O |  |  |
| SNN |  | O |  |  |
| SNRNP48 |  | O |  |  |
| SOCS5 |  | O |  |  |
| SP100 |  | O |  |  |
| SP3 |  | O |  |  |
| SPAG9 |  | O |  |  |
| SPCS3 |  | O |  |  |
| SPN |  | O |  |  |
| SPOCK2 |  | O |  |  |
| SPOPL |  | O |  |  |
| SRCAP |  | O |  |  |
| SRGAP1 |  | O |  |  |
| SRPK1 |  | O |  |  |
| SSH2 |  | O |  |  |
| SSX2IP |  | O |  |  |
| STAC2 |  | O |  |  |
| STK39 |  | O |  |  |
| STRADB |  | O |  |  |
| STRBP |  | O |  |  |
| STX7 |  | O |  |  |
| SULF1 |  | O |  |  |
| SUSD3 |  | O |  |  |
| SYT16 |  | O |  |  |
| SYTL5 |  | O |  |  |
| TAB3 |  | O |  |  |
| TAF1 |  | O |  |  |
| TAF9B |  | O |  |  |
| TANC2 |  | O |  |  |
| TBC1D12 |  | O |  |  |
| TBC1D13 |  | O |  |  |
| TBC1D30 |  | O |  |  |
| TBC1D4 |  | O |  |  |
| TDG |  | O |  |  |
| TDRP |  | O |  |  |
| TFAM |  | O |  |  |
| THAP9 |  | O |  |  |
| THNSL1 |  | O |  |  |
| TLR4 |  | O |  |  |
| TM4SF18 |  | O |  |  |
| TMCC1 |  | O |  |  |
| TMEM106B |  | O |  |  |
| TMEM132C |  | O |  |  |
| TMEM220 |  | O |  |  |
| TMEM236 |  | O |  |  |
| TMEM33 |  | O | O | O |
| TMEM56 |  | O |  |  |
| TMEM64 |  | O |  |  |
| TMEM68 |  | O |  |  |
| TMEM86A |  | O |  |  |
| TMPPE |  | O |  |  |
| TNFRSF19 |  | O |  |  |
| TNKS |  | O |  |  |
| TNPO1 |  | O |  |  |
| TNRC6A |  | O |  |  |
| TNRC6B |  | O |  |  |
| TOB1 |  | O |  |  |
| TPM3 |  | O |  |  |
| TRAPPC2 |  | O |  |  |
| TRERF1 |  | O |  |  |
| TRIP12 |  | O |  |  |
| TRPC3 |  | O |  |  |
| TRPC4 |  | O |  |  |
| TRPM8 |  | O |  |  |
| TTC30A |  | O |  |  |
| TTC39C |  | O |  |  |
| TXNDC16 |  | O |  |  |
| UBE2H |  | O |  |  |
| UBE2K |  | O |  |  |
| UBE4B |  | O |  |  |
| UBR7 |  | O |  |  |
| UGGT1 |  | O |  |  |
| UHMK1 |  | O |  |  |
| UHRF1BP1 |  | O | O | O |
| ULK1 |  | O |  |  |
| ULK2 |  | O |  |  |
| UPRT |  | O |  |  |
| USP37 |  | O |  |  |
| USP49 |  | O |  |  |
| VAPB |  | O |  |  |
| VASP |  | O |  |  |
| WBSCR16 |  | O |  |  |
| WDR20 |  | O |  |  |
| WDR33 |  | O |  |  |
| WDR72 |  | O |  |  |
| WNT9B |  | O |  |  |
| WRB |  | O |  |  |
| XDH |  | O |  |  |
| XK |  | O |  |  |
| XKR6 |  | O |  |  |
| XPR1 |  | O |  |  |
| XRN1 |  | O |  |  |
| YPEL1 |  | O |  |  |
| ZAK |  | O |  |  |
| ZBTB10 |  | O |  |  |
| ZBTB24 |  | O |  |  |
| ZBTB33 |  | O |  |  |
| ZBTB45 |  | O |  |  |
| ZDHHC18 |  | O |  |  |
| ZDHHC6 |  | O |  |  |
| ZFHX3 |  | O |  |  |
| ZNF106 |  | O |  |  |
| ZNF12 |  | O |  |  |
| ZNF200 |  | O |  |  |
| ZNF212 |  | O |  |  |
| ZNF24 |  | O |  |  |
| ZNF260 |  | O |  |  |
| ZNF37A |  | O |  |  |
| ZNF462 |  | O |  |  |
| ZNF469 |  | O |  |  |
| ZNF547 |  | O |  |  |
| ZNF594 |  | O |  |  |
| ZNF608 |  | O |  |  |
| ZNF710 |  | O |  |  |
| ZNF772 |  | O |  |  |
| ZNF81 |  | O |  |  |
| ADAP2 |  |  | O |  |
| ADM2 |  |  | O |  |
| AKAP6 |  |  | O |  |
| ANKH |  |  | O |  |
| AP1G1 |  |  | O |  |
| ARHGEF6 |  |  | O |  |
| ARMC10 |  |  | O |  |
| ASB8 |  |  | O |  |
| ATF7IP |  |  | O |  |
| ATP9A |  |  | O |  |
| BSG |  |  | O |  |
| C19orf44 |  |  | O |  |
| C1orf198 |  |  | O |  |
| CAMLG |  |  | O |  |
| CENPBD1 |  |  | O |  |
| CEP19 |  |  | O |  |
| CHRNA5 |  |  | O |  |
| CYTH2 |  |  | O |  |
| DNAJC5 |  |  | O |  |
| EREG |  |  | O |  |
| FAM189A1 |  |  | O |  |
| FDX1 |  |  | O |  |
| FKBP5 |  |  | O |  |
| FOXK1 |  |  | O |  |
| GAS7 |  |  | O |  |
| GCM1 |  |  | O |  |
| GEN1 |  |  | O |  |
| GTPBP10 |  |  | O |  |
| IGF2BP2 |  |  | O |  |
| LAT2 |  |  | O |  |
| LRRC14 |  |  | O |  |
| LRRN4CL |  |  | O |  |
| MAPKBP1 |  |  | O |  |
| MEGF9 |  |  | O |  |
| MINOS1 |  |  | O |  |
| MPP2 |  |  | O |  |
| MTMR1 |  |  | O |  |
| MUC4 |  |  | O |  |
| MYO6 |  |  | O |  |
| NADK |  |  | O |  |
| NF2 |  |  | O |  |
| NKX2-3 |  |  | O |  |
| NMT1 |  |  | O |  |
| NXF1 |  |  | O |  |
| PDDC1 |  |  | O |  |
| PER2 |  |  | O |  |
| PLEKHA2 |  |  | O |  |
| PLIN3 |  |  | O |  |
| PNPT1 |  |  | O |  |
| PPIL2 |  |  | O |  |
| PRPF4 |  |  | O |  |
| PSKH1 |  |  | O |  |
| QSOX1 |  |  | O |  |
| RAPGEF1 |  |  | O |  |
| SAR1B |  |  | O |  |
| SCPEP1 |  |  | O |  |
| SERF1A |  |  | O |  |
| SERF1B |  |  | O |  |
| SEZ6 |  |  | O |  |
| SIRPB2 |  |  | O |  |
| SIT1 |  |  | O |  |
| SLC31A1 |  |  | O |  |
| SNAP29 |  |  | O |  |
| SRM |  |  | O |  |
| SRRM4 |  |  | O |  |
| STK35 |  |  | O |  |
| STK4 |  |  | O |  |
| TGM5 |  |  | O |  |
| TNFRSF10D |  |  | O |  |
| TRAF3IP1 |  |  | O |  |
| TRIM35 |  |  | O |  |
| TTPAL |  |  | O |  |
| VPS41 |  |  | O |  |
| VPS4B |  |  | O |  |
| WHAMM |  |  | O |  |
| WWC1 |  |  | O |  |
| XPNPEP3 |  |  | O |  |
| YPEL2 |  |  | O |  |
| ZDHHC23 |  |  | O |  |

**Supplementary Table 5. Functionally enriched terms in target genes of the three miRNA markers**

# FDR: False Dicovery Rate, B&H: Benjamini–Hochberg procedure, B&Y: Benjamini–Hochberg–Yekutieli procedure

| Category | ID | Name | p-value | q-value Bonferroni | q-value FDR B&H | q-value FDR B&Y | Hit Count in Query List | Hit Count in Genome |
| --- | --- | --- | --- | --- | --- | --- | --- | --- |
| GO: Molecular Function | GO:0019899 | enzyme binding | 1.43E-12 | 2.55E-09 | 2.55E-09 | 2.05E-08 | 200 | 1933 |
| GO: Molecular Function | GO:0016773 | phosphotransferase activity, alcohol group as acceptor | 4.58E-07 | 8.17E-04 | 3.02E-04 | 2.44E-03 | 86 | 781 |
| GO: Molecular Function | GO:0004674 | protein serine/threonine kinase activity | 6.47E-07 | 1.15E-03 | 3.02E-04 | 2.44E-03 | 57 | 452 |
| GO: Molecular Function | GO:0004672 | protein kinase activity | 8.03E-07 | 1.43E-03 | 3.02E-04 | 2.44E-03 | 74 | 649 |
| GO: Molecular Function | GO:0016301 | kinase activity | 8.46E-07 | 1.51E-03 | 3.02E-04 | 2.44E-03 | 91 | 853 |
| GO: Molecular Function | GO:0019904 | protein domain specific binding | 2.94E-06 | 5.25E-03 | 8.75E-04 | 7.06E-03 | 79 | 732 |
| GO: Molecular Function | GO:0032553 | ribonucleotide binding | 5.14E-06 | 9.17E-03 | 1.28E-03 | 1.03E-02 | 167 | 1882 |
| GO: Molecular Function | GO:0016772 | transferase activity, transferring phosphorus-containing groups | 5.73E-06 | 1.02E-02 | 1.28E-03 | 1.03E-02 | 100 | 1006 |
| GO: Molecular Function | GO:0032555 | purine ribonucleotide binding | 7.23E-06 | 1.29E-02 | 1.43E-03 | 1.16E-02 | 165 | 1866 |
| GO: Molecular Function | GO:0019900 | kinase binding | 8.83E-06 | 1.58E-02 | 1.58E-03 | 1.27E-02 | 74 | 693 |
| GO: Molecular Function | GO:0017076 | purine nucleotide binding | 1.14E-05 | 2.03E-02 | 1.64E-03 | 1.32E-02 | 165 | 1881 |
| GO: Molecular Function | GO:0032550 | purine ribonucleoside binding | 1.26E-05 | 2.24E-02 | 1.64E-03 | 1.32E-02 | 161 | 1830 |
| GO: Molecular Function | GO:0035639 | purine ribonucleoside triphosphate binding | 1.28E-05 | 2.28E-02 | 1.64E-03 | 1.32E-02 | 160 | 1817 |
| GO: Molecular Function | GO:0032549 | ribonucleoside binding | 1.37E-05 | 2.45E-02 | 1.64E-03 | 1.32E-02 | 161 | 1833 |
| GO: Molecular Function | GO:0001883 | purine nucleoside binding | 1.37E-05 | 2.45E-02 | 1.64E-03 | 1.32E-02 | 161 | 1833 |
| GO: Molecular Function | GO:0001882 | nucleoside binding | 1.69E-05 | 3.02E-02 | 1.89E-03 | 1.52E-02 | 161 | 1840 |
| GO: Biological Process | GO:0006468 | protein phosphorylation | 6.84E-11 | 5.22E-07 | 5.22E-07 | 4.97E-06 | 195 | 1954 |
| GO: Biological Process | GO:0022008 | neurogenesis | 2.34E-09 | 1.79E-05 | 6.34E-06 | 6.04E-05 | 164 | 1638 |
| GO: Biological Process | GO:0048699 | generation of neurons | 2.49E-09 | 1.90E-05 | 6.34E-06 | 6.04E-05 | 156 | 1538 |
| GO: Biological Process | GO:0030030 | cell projection organization | 4.93E-09 | 3.76E-05 | 9.40E-06 | 8.95E-05 | 146 | 1427 |
| GO: Biological Process | GO:0042325 | regulation of phosphorylation | 2.29E-08 | 1.75E-04 | 2.78E-05 | 2.65E-04 | 150 | 1512 |
| GO: Biological Process | GO:2000026 | regulation of multicellular organismal development | 2.36E-08 | 1.81E-04 | 2.78E-05 | 2.65E-04 | 180 | 1901 |
| GO: Biological Process | GO:0031175 | neuron projection development | 2.55E-08 | 1.95E-04 | 2.78E-05 | 2.65E-04 | 105 | 956 |
| GO: Biological Process | GO:0030182 | neuron differentiation | 3.15E-08 | 2.40E-04 | 2.95E-05 | 2.81E-04 | 141 | 1405 |
| GO: Biological Process | GO:0048666 | neuron development | 3.48E-08 | 2.66E-04 | 2.95E-05 | 2.81E-04 | 118 | 1120 |
| GO: Biological Process | GO:1902531 | regulation of intracellular signal transduction | 7.18E-08 | 5.48E-04 | 5.00E-05 | 4.76E-04 | 169 | 1786 |
| GO: Biological Process | GO:0051174 | regulation of phosphorus metabolic process | 7.20E-08 | 5.50E-04 | 5.00E-05 | 4.76E-04 | 168 | 1773 |
| GO: Biological Process | GO:0006928 | movement of cell or subcellular component | 1.04E-07 | 7.93E-04 | 6.42E-05 | 6.11E-04 | 176 | 1888 |
| GO: Biological Process | GO:0032989 | cellular component morphogenesis | 1.09E-07 | 8.35E-04 | 6.42E-05 | 6.11E-04 | 141 | 1434 |
| GO: Biological Process | GO:0051270 | regulation of cellular component movement | 1.89E-07 | 1.44E-03 | 9.85E-05 | 9.37E-04 | 94 | 861 |
| GO: Biological Process | GO:0019220 | regulation of phosphate metabolic process | 1.93E-07 | 1.48E-03 | 9.85E-05 | 9.37E-04 | 165 | 1760 |
| GO: Biological Process | GO:0000902 | cell morphogenesis | 2.74E-07 | 2.09E-03 | 1.26E-04 | 1.20E-03 | 132 | 1341 |
| GO: Biological Process | GO:0001932 | regulation of protein phosphorylation | 2.85E-07 | 2.18E-03 | 1.26E-04 | 1.20E-03 | 137 | 1406 |
| GO: Biological Process | GO:0045595 | regulation of cell differentiation | 2.98E-07 | 2.27E-03 | 1.26E-04 | 1.20E-03 | 160 | 1706 |
| GO: Biological Process | GO:0018209 | peptidyl-serine modification | 4.39E-07 | 3.35E-03 | 1.76E-04 | 1.68E-03 | 41 | 278 |
| GO: Biological Process | GO:0000904 | cell morphogenesis involved in differentiation | 4.91E-07 | 3.75E-03 | 1.88E-04 | 1.79E-03 | 91 | 843 |
| GO: Biological Process | GO:0007420 | brain development | 5.20E-07 | 3.97E-03 | 1.89E-04 | 1.80E-03 | 84 | 760 |
| GO: Biological Process | GO:0060429 | epithelium development | 5.57E-07 | 4.25E-03 | 1.93E-04 | 1.84E-03 | 128 | 1307 |
| GO: Biological Process | GO:0044093 | positive regulation of molecular function | 6.22E-07 | 4.75E-03 | 2.06E-04 | 1.96E-03 | 173 | 1899 |
| GO: Biological Process | GO:0007417 | central nervous system development | 6.82E-07 | 5.21E-03 | 2.06E-04 | 1.96E-03 | 104 | 1009 |
| GO: Biological Process | GO:0007264 | small GTPase mediated signal transduction | 6.96E-07 | 5.32E-03 | 2.06E-04 | 1.96E-03 | 69 | 589 |
| GO: Biological Process | GO:0009719 | response to endogenous stimulus | 7.03E-07 | 5.36E-03 | 2.06E-04 | 1.96E-03 | 161 | 1743 |
| GO: Biological Process | GO:0018105 | peptidyl-serine phosphorylation | 7.98E-07 | 6.09E-03 | 2.26E-04 | 2.15E-03 | 39 | 264 |
| GO: Biological Process | GO:0071495 | cellular response to endogenous stimulus | 8.87E-07 | 6.78E-03 | 2.42E-04 | 2.30E-03 | 125 | 1280 |
| GO: Biological Process | GO:0060322 | head development | 9.33E-07 | 7.12E-03 | 2.46E-04 | 2.34E-03 | 87 | 807 |
| GO: Biological Process | GO:0048729 | tissue morphogenesis | 1.36E-06 | 1.04E-02 | 3.29E-04 | 3.13E-03 | 83 | 766 |
| GO: Biological Process | GO:0072359 | circulatory system development | 1.38E-06 | 1.05E-02 | 3.29E-04 | 3.13E-03 | 107 | 1062 |
| GO: Biological Process | GO:0072358 | cardiovascular system development | 1.38E-06 | 1.05E-02 | 3.29E-04 | 3.13E-03 | 107 | 1062 |
| GO: Biological Process | GO:0023014 | signal transduction by protein phosphorylation | 1.44E-06 | 1.10E-02 | 3.33E-04 | 3.17E-03 | 99 | 963 |
| GO: Biological Process | GO:2000145 | regulation of cell motility | 1.66E-06 | 1.27E-02 | 3.73E-04 | 3.55E-03 | 85 | 794 |
| GO: Biological Process | GO:0007409 | axonogenesis | 1.78E-06 | 1.36E-02 | 3.88E-04 | 3.69E-03 | 58 | 478 |
| GO: Biological Process | GO:0043408 | regulation of MAPK cascade | 1.88E-06 | 1.44E-02 | 3.99E-04 | 3.80E-03 | 80 | 736 |
| GO: Biological Process | GO:0061564 | axon development | 2.29E-06 | 1.75E-02 | 4.72E-04 | 4.49E-03 | 61 | 516 |
| GO: Biological Process | GO:0040011 | locomotion | 2.49E-06 | 1.90E-02 | 5.00E-04 | 4.76E-03 | 158 | 1740 |
| GO: Biological Process | GO:0060284 | regulation of cell development | 2.66E-06 | 2.03E-02 | 5.21E-04 | 4.95E-03 | 100 | 989 |
| GO: Biological Process | GO:0002009 | morphogenesis of an epithelium | 2.91E-06 | 2.22E-02 | 5.52E-04 | 5.25E-03 | 69 | 613 |
| GO: Biological Process | GO:0042327 | positive regulation of phosphorylation | 2.96E-06 | 2.26E-02 | 5.52E-04 | 5.25E-03 | 101 | 1004 |
| GO: Biological Process | GO:0045935 | positive regulation of nucleobase-containing compound metabolic process | 3.06E-06 | 2.33E-02 | 5.56E-04 | 5.29E-03 | 165 | 1840 |
| GO: Biological Process | GO:0043410 | positive regulation of MAPK cascade | 3.33E-06 | 2.54E-02 | 5.92E-04 | 5.63E-03 | 61 | 522 |
| GO: Biological Process | GO:0048667 | cell morphogenesis involved in neuron differentiation | 3.56E-06 | 2.72E-02 | 6.09E-04 | 5.80E-03 | 67 | 593 |
| GO: Biological Process | GO:0051173 | positive regulation of nitrogen compound metabolic process | 3.59E-06 | 2.74E-02 | 6.09E-04 | 5.80E-03 | 173 | 1953 |
| GO: Biological Process | GO:0051960 | regulation of nervous system development | 4.04E-06 | 3.09E-02 | 6.71E-04 | 6.38E-03 | 91 | 886 |
| GO: Biological Process | GO:0048812 | neuron projection morphogenesis | 4.60E-06 | 3.51E-02 | 7.47E-04 | 7.11E-03 | 70 | 633 |
| GO: Biological Process | GO:0006357 | regulation of transcription from RNA polymerase II promoter | 4.84E-06 | 3.69E-02 | 7.69E-04 | 7.32E-03 | 170 | 1922 |
| GO: Biological Process | GO:0030334 | regulation of cell migration | 5.02E-06 | 3.83E-02 | 7.71E-04 | 7.33E-03 | 79 | 743 |
| GO: Biological Process | GO:0043085 | positive regulation of catalytic activity | 5.06E-06 | 3.86E-02 | 7.71E-04 | 7.33E-03 | 145 | 1588 |
| GO: Biological Process | GO:0032486 | Rap protein signal transduction | 5.15E-06 | 3.93E-02 | 7.71E-04 | 7.33E-03 | 7 | 13 |
| GO: Biological Process | GO:0045860 | positive regulation of protein kinase activity | 5.67E-06 | 4.33E-02 | 8.21E-04 | 7.81E-03 | 58 | 496 |
| GO: Biological Process | GO:0016477 | cell migration | 5.71E-06 | 4.36E-02 | 8.21E-04 | 7.81E-03 | 123 | 1302 |
| GO: Biological Process | GO:0031399 | regulation of protein modification process | 5.85E-06 | 4.46E-02 | 8.21E-04 | 7.81E-03 | 164 | 1847 |
| GO: Biological Process | GO:0035239 | tube morphogenesis | 5.91E-06 | 4.52E-02 | 8.21E-04 | 7.81E-03 | 51 | 417 |
| GO: Cellular Component | GO:0030054 | cell junction | 1.08E-10 | 9.22E-08 | 9.22E-08 | 6.75E-07 | 133 | 1202 |
| GO: Cellular Component | GO:0005911 | cell-cell junction | 2.31E-07 | 1.97E-04 | 9.84E-05 | 7.21E-04 | 55 | 420 |
| GO: Cellular Component | GO:0031252 | cell leading edge | 7.07E-06 | 6.04E-03 | 2.01E-03 | 1.48E-02 | 48 | 389 |
| GO: Cellular Component | GO:0070161 | anchoring junction | 1.39E-05 | 1.18E-02 | 2.96E-03 | 2.17E-02 | 57 | 503 |
| GO: Cellular Component | GO:0005912 | adherens junction | 1.80E-05 | 1.54E-02 | 3.08E-03 | 2.26E-02 | 55 | 484 |
| GO: Cellular Component | GO:0030426 | growth cone | 3.08E-05 | 2.63E-02 | 4.02E-03 | 2.94E-02 | 27 | 182 |
| GO: Cellular Component | GO:0061695 | transferase complex, transferring phosphorus-containing groups | 3.29E-05 | 2.81E-02 | 4.02E-03 | 2.94E-02 | 33 | 245 |
| GO: Cellular Component | GO:0030427 | site of polarized growth | 5.00E-05 | 4.27E-02 | 4.95E-03 | 3.62E-02 | 27 | 187 |
| GO: Cellular Component | GO:1990234 | transferase complex | 5.21E-05 | 4.45E-02 | 4.95E-03 | 3.62E-02 | 73 | 724 |
| Mouse Phenotype | MP:0001525 | impaired balance | 1.67E-06 | 1.00E-02 | 5.10E-03 | 4.73E-02 | 29 | 145 |
| Mouse Phenotype | MP:0009956 | abnormal cerebellar layer morphology | 1.70E-06 | 1.02E-02 | 5.10E-03 | 4.73E-02 | 49 | 315 |
| Mouse Phenotype | MP:0001516 | abnormal motor coordination/ balance | 4.65E-06 | 2.79E-02 | 6.96E-03 | 6.45E-02 | 100 | 836 |
| Mouse Phenotype | MP:0000877 | abnormal Purkinje cell morphology | 4.91E-06 | 2.95E-02 | 6.96E-03 | 6.45E-02 | 38 | 228 |
| Mouse Phenotype | MP:0008572 | abnormal Purkinje cell dendrite morphology | 6.36E-06 | 3.81E-02 | 6.96E-03 | 6.45E-02 | 18 | 72 |
| Mouse Phenotype | MP:0002127 | abnormal cardiovascular system morphology | 6.96E-06 | 4.17E-02 | 6.96E-03 | 6.45E-02 | 199 | 1954 |
| Pathway | 105688 | Axon guidance | 5.77E-09 | 1.25E-05 | 1.25E-05 | 1.03E-04 | 43 | 262 |
| Pathway | 477129 | Developmental Biology | 6.06E-08 | 1.31E-04 | 6.54E-05 | 5.40E-04 | 56 | 419 |
| Pathway | 198845 | Insulin Signaling | 3.39E-06 | 7.32E-03 | 2.44E-03 | 2.01E-02 | 27 | 163 |
| Pathway | 782000 | Proteoglycans in cancer | 4.60E-06 | 9.94E-03 | 2.48E-03 | 2.05E-02 | 33 | 225 |
| Pathway | 198909 | B Cell Receptor Signaling Pathway | 1.97E-05 | 4.26E-02 | 8.29E-03 | 6.84E-02 | 25 | 159 |
| Pathway | 119527 | Signaling by Robo receptor | 2.30E-05 | 4.97E-02 | 8.29E-03 | 6.84E-02 | 10 | 33 |
| Disease | umls:C3714756 | Intellectual Disability | 4.55E-08 | 2.57E-04 | 2.57E-04 | 2.37E-03 | 54 | 413 |
| Disease | umls:C0014175 | Endometriosis | 4.47E-07 | 2.52E-03 | 1.02E-03 | 9.38E-03 | 72 | 659 |
| Disease | umls:C0699790 | Colon Carcinoma | 6.85E-07 | 3.87E-03 | 1.02E-03 | 9.38E-03 | 160 | 1854 |
| Disease | umls:C0025362 | Mental Retardation | 7.21E-07 | 4.07E-03 | 1.02E-03 | 9.38E-03 | 50 | 403 |
| Disease | umls:C0017636 | Glioblastoma | 1.05E-06 | 5.94E-03 | 1.19E-03 | 1.09E-02 | 140 | 1584 |
| Disease | umls:C1762616 | Meningioma, benign, no ICD-O subtype | 1.98E-06 | 1.12E-02 | 1.86E-03 | 1.71E-02 | 32 | 217 |
| Disease | umls:C0014544 | Epilepsy | 2.79E-06 | 1.58E-02 | 2.13E-03 | 1.96E-02 | 62 | 567 |
| Disease | umls:C0007102 | Malignant tumor of colon | 3.02E-06 | 1.70E-02 | 2.13E-03 | 1.96E-02 | 147 | 1714 |
| Disease | umls:C0004936 | Mental disorders | 4.12E-06 | 2.32E-02 | 2.38E-03 | 2.20E-02 | 43 | 346 |
| Disease | umls:C0178874 | Tumor Progression | 4.22E-06 | 2.38E-02 | 2.38E-03 | 2.20E-02 | 164 | 1969 |
| Disease | umls:C0025286 | Meningioma | 4.85E-06 | 2.73E-02 | 2.48E-03 | 2.29E-02 | 46 | 383 |
| Disease | umls:C0023267 | Fibroid Tumor | 8.30E-06 | 4.68E-02 | 3.84E-03 | 3.54E-02 | 37 | 287 |
